# Cultivation of Important Methanotrophs From Indian Rice Fields

**DOI:** 10.3389/fmicb.2021.669244

**Published:** 2021-09-03

**Authors:** Monali C. Rahalkar, Kumal Khatri, Pranitha Pandit, Rahul A. Bahulikar, Jyoti A. Mohite

**Affiliations:** ^1^C2, Bioenergy Group, MACS Agharkar Research Institute, Pune, India; ^2^Department of Microbiology, Savitribai Phule Pune University, Pune, India; ^3^Central Research Station, BAIF Development Research Foundation, Pune, India

**Keywords:** methane, novel, India, rice fields, cultivation, metagenomics, methanotrophs, serial dilution

## Abstract

Methanotrophs are aerobic to micro-aerophilic bacteria, which oxidize and utilize methane, the second most important greenhouse gas. The community structure of the methanotrophs in rice fields worldwide has been studied mainly using culture-independent methods. Very few studies have focused on culturing methanotrophs from rice fields. We developed a unique method for the cultivation of methanotrophs from rice field samples. Here, we used a modified dilute nitrate mineral salts (dNMS) medium, with two cycles of dilution till extinction series cultivation with prolonged incubation time, and used agarose in the solid medium. The cultivation approach resulted in the isolation of methanotrophs from seven genera from the three major groups: Type Ia (*Methylomonas*, *Methylomicrobium*, and *Methylocucumis*), Type Ib (*Methylocaldum* and *Methylomagnum*), and Type II (*Methylocystis* and *Methylosinus*). Growth was obtained till 10^–6^–10^–8^ dilutions in the first dilution series, indicating the culturing of dominant methanotrophs. Our study was supported by 16S rRNA gene-based next-generation sequencing (NGS) of three of the rice samples. Our analyses and comparison with the global scenario suggested that the cultured members represented the major detected taxa. Strain RS1, representing a putative novel species of *Methylomicrobium*, was cultured; and the draft genome sequence was obtained. Genome analysis indicated that RS1 represented a new putative *Methylomicrobium* species. *Methylomicrobium* has been detected globally in rice fields as a dominant genus, although no *Methylomicrobium* strains have been isolated from rice fields worldwide. Ours is one of the first extensive studies on cultured methanotrophs from Indian rice fields focusing on the tropical region, and a unique method was developed. A total of 29 strains were obtained, which could be used as models for studying methane mitigation from rice fields and for environmental and biotechnological applications.

## Introduction

Methane is the second most important greenhouse gas after carbon dioxide. Aerobic methanotrophs use methane gas as their sole source of carbon and energy source (Hanson and Hanson, 1996). Most of the described and available methanotrophs belong to the phylum Proteobacteria. Methanotrophs belonging to the phylum Verrucomicrobia (Pol et al., 2007) and Candidatus phylum NC-10 have also been described in recent years (Ettwig et al., 2010; Dedysh and Knief, 2018). The proteobacterial methanotrophs are classified as Type I (Gammaproteobacteria) and Type II (Alphaproteobacteria) based on the internal cell structure, phylogeny, and carbon assimilation pathway. Methanotrophs inhabit rice fields (Frenzel, 2012), lakes (Pester et al., 2004; Rahalkar, 2007; Oswald et al., 2016), wetlands (Dedysh, 2009), soda lakes (Antony et al., 2012), compost (Kim et al., 2008), forest soils (Dunfield et al., 2003), peat bogs (Pytlak et al., 2012), thermal springs (Hirayama et al., 2011), and marine sediments (Bowman et al., 1997; Tavormina et al., 2015).

The scholarly work of Whittenbury et al. (1970) started a new era of cultured methanotrophs in 1970. In the last 50 years, several new genera and species have been documented, and a large proportion of the diversity of aerobic methanotrophs has been brought into culture. However, several lineages lack cultivated representatives and pose challenges for further cultivation studies (Dedysh and Dunfield, 2014; Dedysh and Knief, 2018). Cultivation of indigenous methanotrophs is crucial, and studies targeting their metabolism, physiology, and biochemistry would help understand them in detail (Bowman, 2011). Methanotrophs can also produce industrially important biomolecules and can be used to produce methanol by using methane as a feedstock (Patel et al., 2018b, 2019, 2020a, 2020b). Biomolecules such as single-cell protein, biopolymers, methanol, formaldehyde, organic acids, ectoine, lipids (biodiesel and health supplements), growth media, and vitamin B12 (Oremland and Culbertson, 1992; Al Hasin et al., 2010; Strong et al., 2015; Patel et al., 2018b) can be produced using methanotrophs using methane.

Rice fields form an important niche for aerobic methanotrophs, where they oxidize about 20% of the methane produced (Conrad, 2009). Both Type I (gammaproteobacterial) and Type II (alphaproteobacterial) methanotrophs are present in the rice rhizosphere (Qiu et al., 2008; Shrestha et al., 2008; Reim et al., 2012; Shiau et al., 2018). Culture-independent analysis of rice field samples showed the following methanotrophs to be present: *Methylobacter*, *Methylomicrobium*, and *Methylomonas* (Type Ia); *Methylocaldum*, *Methylococcus*, and uncultured (Type Ib); and *Methylocystis* (Type II) (Noll et al., 2008; Lüke et al., 2010; Ma et al., 2013). However, very few members have been cultured, and several *pmoA* lineages, rice paddy cluster (e.g., RPC1–3), detected remain uncultured (Lüke, 2010; Knief, 2015). Only a handful of cultivation-based studies worldwide have targeted the cultivation of methanotrophs from rice fields (Dianou et al., 2009; Ferrando and Tarlera, 2009; Pandit et al., 2016; Frindte et al., 2017). In each of documented studies, members of up to four genera have been cultured.

Here, we report extensive efforts taken to culture dominantly present methanotrophs from Indian rice fields. We describe here a method that could be used for the enrichment and isolation of methanotrophs from a rice field sample. We used a mineral medium that had been successfully used before for culturing rice field methanotrophs. A first set of dilution till extinction was set up and incubated for a prolonged time for 6–8 weeks. In a second dilution series using microtiter plates, we could achieve isolation of single methanotroph cultures, which were then further purified as pure or axenic cultures by plating on a solid medium and re-streaking. This way, the slow-growing but numerically dominant cultures were given a chance to grow; and many pure cultures were obtained as a result. Additionally, we used 16S rRNA gene-based next-generation sequencing (NGS) to document the actual diversity of methanotrophs present in a few samples.

## Material and Equipment

### Growth Media

(1) Nitrate mineral salts (NMS) medium (Whittenbury et al., 1970) g/L: MgSO_4_ 7H_2_O, 1; CaCl_2_ 2H_2_O, 0.2; KNO_3_, 1; SL10 solution, 1 ml; Fe_3_NH_4_ Citrate solution, 1 ml.

Post autoclave additions: phosphate buffer (pH 6.8), 20 ml/L and vitamin solution (1×), 10 ml/L.

(2) Modified dilute NMS (dNMS) medium (Pandit et al., 2016) g/L: MgSO_4_ 7H_2_O, 1; CaCl_2_ 2H_2_O, 0.2; KNO_3_, 0.25; SL10 solution, 1 ml; Fe_3_NH_4_ citrate solution, 1 ml; HEPES buffer (2 M, pH 7), 1 ml.

Post autoclave additions: phosphate buffer (pH 6.8), 2 ml/L, and vitamin solution (1×), 10 ml/L.

(3) Solidifying agent: 2% agarose.

(4) Stock solution compositions are as follows:

(a) SL10 (trace element solution) mg/L: ZnCl_2_, 70; MnCl_2_ 2H_2_O, 100; Na_2_MoO_4_ 2H_2_O, 36; CuCl_2_ 2H_2_O, 17; ^∗^FeCl_4_ 4H_2_O, 1,500; CoCl_2_ 6H_2_O, 190; H_3_BO_3_, 62; NiCl_2_ 6H_2_O, 24; ^∗^HCl (25%), 10 ml. Note: Prepare 25% HCl and dissolve 0.15 g/1,500 mg of FeCl_4_ 4H_2_O in 1 ml of 25% HCl.

(b) Fe_3_NH_4_ citrate: 0.2 g/50 ml of distilled water.

(c) Phosphate buffer, pH 6.8 (g/L): Na_2_HPO_4_, 3.6, and KH_2_PO_4_, 1.4 g/L addition. Autoclaved phosphate buffer stock solution was stored at 4–8°C (refrigeration).

(d) Vitamin solution (1×): chemicals dissolved as mg/L: biotin, 2; folic acid, 2; pyridoxine-HCl, 10; thiamine-HCl 2H_2_O, 5; riboflavin, 5; nicotinic acid, 5; D-Ca-pantothenate, 5; vitamin B12, 20; *p*-aminobenzoic acid, 5; lipoic acid, 5; nicotinamide, 5; pyridoxal HCl, 5; and L-ascorbic acid, 5 mg per liter.

Phosphate buffer and vitamin stock solutions were sterilized separately.

All the chemicals or reagents were of analytical grade of the brands Sisco Research Laboratories Pvt. Ltd. (SRL) or Sigma Aldrich Chemicals Private Limited.

### Cultivation Material

(1) Serum bottles—65- or 35-ml capacity.

(2) Silicone butyl rubber stoppers, aluminum crimp seals, and a bottle crimper.

(3) Glass desiccators of 25-L volume.

### Microscopy

(1) Phase-contrast microscope, Nikon 80i, Japan microscope with 10, 40, and 100× (oil emersion lens) objective lenses.

(2) Scanning electron microscopy (SEM) (Zeiss model EVO-MA-15 SEM).

(3) Slides for phase-contrast microscopy and glass coverslips.

### DNA Extraction

(1) Bacterial DNA extraction—Sigma GenElute^TM^ *Bacterial Genomic DNA Kit* (*Gram-negative protocol*).

(2) Soil DNA extraction—MP Biomedicals FastDNA^TM^ Spin Kit for Soil DNA Extraction.

### Methane Oxidation Measure

Chemito 8510 Gas Chromatograph, India, equipped with a flame ionization detector (FID). Conditions: column PorapakQ (80/100 mesh size, 3.2 mm × 2 mm); injector temperature 110°C; detector temperature 120°C; oven temperature 100°C; flow rates of carrier gasses, nitrogen (15–18 ml/min), hydrogen (50 ml/min), and air (250 ml/min).

### Molecular Identification

(1) *pmoA* gene primers: 189 forward (5′-GGNGACTGGGACTTCTGG-3′) and 661 reverse (5′-CGGNGCAACGTCYTTACC-3′).

(2) Universal 16S rRNA primers: 27 forward (5′-AGAGTTTGATCMTGGCTCAG-3′) and 1492 reverse (5′-TACGGYTACCTTGTTACGACTT-3′).

## Methods

### Step 1: Sampling

Rice field samples were collected from three geographical locations from Kerala and Maharashtra states in India (Supplementary Table 1). Most of the samples were collected at the end of the monsoon in October. The plants were in the late flowering to grain-bearing stage this month. Due to difficulties in the accessibility of the region in Kerala, where there are heavy rains in the monsoon season, we collected samples from the Kerala rice fields and marshy area in December. Three of the samples (KRF, KM, and KB) were used for the culture-independent analysis of methanotrophs using 16S rRNA gene-based NGS targeting the V3–V4 region. Cultivation of methanotrophs was done from all five locations (Supplementary Table 1). The sampling was done as described before (Pandit et al., 2016), where pooled soil samples from three to five distantly located rice plants from each rice field were used. The rice plants were uprooted with the entire root system and the attached soil. Only rhizospheric soil was collected by removing the bulk soil and scraping the soil attached to the roots. In one case, soil attached to the stem (1–3 cm from the roots) was collected. The samples from Kerala were collected in sterile sealed gas-tight bags and brought to the lab in the shortest possible time (3–4 days). All the samples were immediately transported to the laboratory on the same day and were processed as detailed earlier (Pandit et al., 2016). The samples were immediately used for enrichment experiments, some portion was stored at −20°C for DNA extraction, and the remaining samples were stored at 4–8°C for further experimentation.

### Step 2: Enrichment and Isolation

**FIGURE 1:**
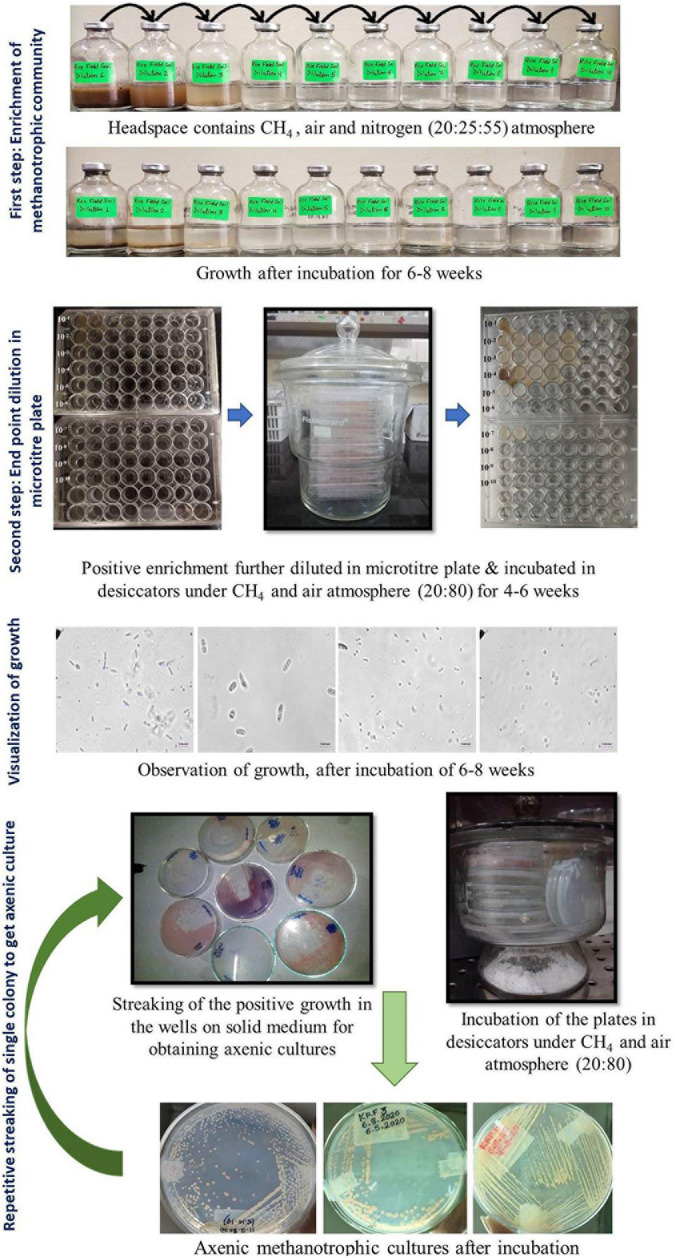
Cultivation approach used in the present study for the enrichment and isolation of methanotrophs using a two-step enrichment.

The entire procedure for the enrichment and isolation of methanotrophs is shown in a flow chart (Figure 1).

#### Enrichment of Methanotrophic Community

1. The modified dNMS medium was prepared as described previously (Pandit et al., 2016). The salts (MgSO_4_⋅7H_2_O, KNO_3_, and CaCl_2_) were dissolved in distilled water as per given in *Growth Media*, part 2.
2. The medium was boiled to remove dissolved oxygen and cooled under nitrogen gas flushing conditions under a gassing manifold.
3. After being cooled, SL-10 trace element solution, 0.4% (w/v) ferric ammonium citrate solution, and 2 M of HEPES buffer (pH 6.8) were added.
4. The pH was adjusted to 6.8 using 5 M of NaOH or KOH.
5. Serum bottles (65-ml volume) were filled with 18 ml of sterile dilute (dNMS) medium (Pandit et al., 2016), and headspace was flushed with nitrogen gas and autoclaved.
6. After being cooled, phosphate buffer and vitamins were added.
7. The samples were serially diluted from 10^–1^ to 10^–10^ by adding 2 ml or 2 g of sample to an 18 ml sterile (dNMS) medium (*Growth Media*, part 2).
8. A headspace volume of 45% was removed with a sterile syringe and filled with 20% methane and 25% air. All the gasses were added using a syringe and a needle through a sterile filter (0.2 μm).
9. The bottles were incubated under static conditions and 25°C in the dark.
10. Methane oxidation was checked periodically using Chemito 8510 Gas Chromatography (Mumbai, India), equipped with a FID.
11. The bottles were incubated for ∼6–8 weeks, and the gas phase was replaced after every 10 days with the same gas mixture as described above.
12. Growth was recorded in the form of turbidity, flakes, or pellets. A simultaneous decline in methane accompanied by growth in the form of visual turbidity, pellicle, or biofilm at the bottom was considered an indication of methanotrophic growth and considered a positive enrichment.

#### End-Point Dilution in Microtiter Plate

Positive methanotroph enrichment of 100 μl was diluted in sterile 900 μl of dNMS media added to a 48-well microtiter plate, and dilution up to 10^–8^ was done. The microtiter plates were placed in a glass airtight desiccator under methane:air environment (20:80) and incubated at 25°C for 4–8 weeks.

#### Visualization of Growth

1. Wet mounts were observed under a phase-contrast microscope Nikon 80i (Tokyo, Japan) microscope under 1, 40, and 100× (oil emersion lens) objective lenses.
2. Cells growing in the enriched liquid in microtiter plates were observed.
3. After growth in the wells was visualized, 50–100 μl of the liquid was streaked onto 2% agarose dNMS solid media plates and incubated in desiccators under methane:air environment (20:80) and incubated at 25°C for 4–6 weeks.
4. The desiccators were opened and checked for growth after every week (Figure 1).

#### Repetitive Streaking of Single Colony to Get Axenic Culture

1. Each isolated colony growing on the agarose medium, which was incubated in the presence of methane, was picked up using sterile toothpicks or wire loops. The cells from every unique colony were observed and re-streaked for purification.
2. Wet mounts from all single colonies appearing on plates incubated under methane were visualized under a phase-contrast microscope (Nikon) equipped with a computer, and images were taken.
3. After several rounds of re-streaking, pure methanotrophic isolates were obtained.

#### Purity Check

The purity of each methanotroph was checked by streaking it on a heterotrophic medium (dilute nutrient agar plus 0.1% glucose or a complete nutrient agar plate). No growth on either of these media was taken as an indication of the purity of the methanotroph.

### Step 3: Morphological Characterization

The morphological characteristics such as colony color, colony morphology, and liquid growth characteristics (pellicle, turbidity, or biofilm) were recorded. Each culture was grown in liquid dNMS medium in 65-ml-capacity serum bottles with 20% methane in the headspace. The decline in methane was followed over time. Each pure culture was grown and processed to observe under a SEM (Zeiss, Oberkochen, Germany; model EVO-MA-15 SEM). Sample preparation for SEM has been described before (Pandit et al., 2018). Analysis of methane oxidation potential of representative strains of each genus was carried out, and the corresponding growth in terms of optical density (OD) was measured for a period of 15 days (Supplementary Material).

### Step 4: DNA Extraction, PCR Amplification, and Sequencing

1. After it was confirmed that the cultures oxidized methane and were pure, DNA extraction was done using either liquid culture pellets or colonies picked from a freshly growing plate. DNA extraction from the isolates was done as described before (Pandit et al., 2016).
2. Particulate methane monooxygenase β subunit (*pmoA*) gene amplification and 16S rRNA gene amplification were carried out using A189f-mb661r primers and 27f and 1492r primers as described before (Pandit et al., 2016) using the extracted DNA from pure cultures.
3. The amplified products were sequenced using initially one and then with forward and reverse primers; and First Base Laboratories, Malaysia, did the sequencing.
4. The sequences obtained using both the primers were aligned and assembled using SeqMan (DNASTAR, Lasergene software) and were subjected to BLAST analysis [sequences of all of the unique isolates were deposited in the National Center for Biotechnology Information (NCBI) database]. If the similarity between the isolates was more than 99.5%, only a single sequence was submitted. Each strain of the methanotroph was classified based on its colony characters, blast using 16S rRNA gene, and *pmoA* partial sequence and assigned to the genus and possible species level.

### Step 5: Whole-Genome Sequencing and Analysis

The whole-genome analysis was carried out as described before (Khatri et al., 2021). The whole-genome sequencing of the culture RS1 was outsourced and done using the Illumina HiSeq platform (151^∗^2) at MedGenome Labs, Bangalore, India. *De novo* assembly of the sequenced reads was done by using SPADES *De novo* Assembler (v3.13.0). The contig files were submitted to RAST^1^, and submitted to the NCBI including the NCBI prokaryotic genome annotation pipeline PGAP^2^. The genome of strain RS1 compared with genomes of its closest members was done to calculate the average nucleotide identity (ANIb-G^3^), digital DNA--DNA hybridization (dDDH^4^), and the average amino acid identity (AAI^5^).

### Step 6: 16S rRNA Next-Generation Sequencing Targeting the V3–V4 Region

1. Metagenomic DNA from the soil samples (KB, KM, and KRF) was extracted using the FAST DNA spin kit for soil (MP Biomedicals, Irvine, CA, United States).
2. After the initial quality was checked using NanoDrop and run on an agarose gel, further 16S rRNA NGS targeting the V3–V4 region was done by Sandor Lifesciences Pvt. Ltd. (Hyderabad, India) (KB and KM samples) and in Helical Lab, Pune (KRF samples).
3. The DNA was amplified using primers specific to the V3–V4 hypervariable region of the 16S rRNA gene.
4. The library was sequenced on an Illumina HiSeq/MiSeq PE 150, i.e., 150 × 2, using paired-end sequencing.
5. Other procedures were done as per the standard operating conditions of each vendor. The companies also performed adaptor trimming, and the read sequence reads specific to the families Methylococcaceae and Methylocystaceae were extracted using Perl script and given to us.
6. Additionally, the data were also checked for the presence of *Methylocella* and *Methylacidiphilum*. These extracted reads from the families Methylococcaceae and Methylocystaceae were used for the phylogenetic tree constructions.
7. The extracted .fas files for each family or genus were blasted in NCBI using nucleotide blast, and the data for abundance for each methanotrophic genus was collected. A cutoff of 95% (16S rDNA fragment) was used for classifying a particular sequence in that genus. The genus and species data were used to calculate the bar diagram ratios for Methylococcaceae and Methylocystaceae.

### Step 7: Phylogenetic Analysis

1. The 16S rRNA sequences from high-throughput sequencing were aligned using MAFFT^6^ and the reference sequences extracted from NCBI^7^.
2. The partial sequences 16S rRNA gene sequences obtained in the NGS were used for the tree construction using the maximum-likelihood bootstrap analysis using other sequences from Methylococcaceae members. Evolutionary analyses were conducted in MEGA7 (Kumar et al., 2016).
3. Similarly, a tree with Methylocystaceae sequences (total of 104 sequences) with average 173 nucleotides in length were used for the construction of a phylogenetic tree using MEGA X (Kumar et al., 2018).
4. A common tree using the 16S rRNA gene sequences of the representative isolates obtained in this study was constructed using maximum-likelihood analysis with 1,000 bootstraps compared with the closely associated members. The phylogenetic trees were constructed using MEGA X and analyzed based on the Tamura–Nei model.
5. The phylogenetic analysis of the putative novel isolate RS1 was done using both the 16S rRNA gene and the partial *pmoA* gene using maximum-likelihood analysis with 1,000 bootstraps compared with the closely associated methanotrophs. The phylogenetic trees were constructed using MEGA X and analyzed based on the Tamura–Nei model.

## Results

### Isolation and Cultivation of Methanotrophs From Seven Genera Using Currently Developed Method

A two-step dilution till extinction enrichment process was used for the cultivation of methanotrophs, where we used serum bottles for the first step followed by serial dilution of each of the positive enrichment in microtiter plates in eight steps (Figure 1). The first enrichment was carried out for 6–8 weeks. Growth was accompanied by methane oxidation, and it was in the form of pellicle, biofilm, or turbidity and was seen up to 10^–1^ to 10^–8^ dilutions in the samples (Table 1). The second dilutions series showed cultures with single morphotypes in the highest dilutions. In most cases, plating the highest dilution from the second series resulted in a mono-culture of a methanotroph after plating. On average, three to four streaking steps resulted in the pure cultures. A total of 29 strains of methanotrophs were cultured in pure form (Table 1). The primary dilution of the sample is indicated for the isolates. Most of the isolates were obtained from the highest dilutions reflecting their dominance in the environment (Table 1). Cells showed large and characteristic cells with coccoid, elliptical, fat, or long rod morphology and mostly showed internal granules. All the cultures exhibited methanotrophy, i.e., grew with methane as the sole source of carbon and energy. All the cultures oxidized methane after an initial lag phase and consumed half in a 5–7 days’ interval and showed a simultaneous growth with an increase in the OD. The growth curves of representative strains showed a maximum increase in the OD of 0.15–0.2 with simultaneous methane oxidation (Supplementary Material). The isolated strains belonged to seven genera after NCBI blast analysis of the 16S rRNA and partial *pmoA* genes (Table 1).

**TABLE 1:** Cultivated methanotroph strains from various samples*.

**Serial number**	**Methanotrophs genus**	**Species/putative novel**	**Total number of isolates, (novel) isolation source**	**Dilution (serum bottle) from which the cultures were isolated**
(1)	***Methylomonas***	koyamae, denitrificans, **methanica**	12**(1)**, KRF, Kb, Kerala mud	10^–2^–10^–5^
(2)	***Methylomicrobium***	**Novel species**	**2 (1), Mulshi**	10^–5^
(3)	*Methylocucumis*	*oryzae*	1 (Mulshi)	10^–5^
(4)	*Methylomagnum*	*ishizawai*	1, KRF	10^–4^
(5)	*Methylocaldum*	*gracile*	1, Mulshi	10^–7^
(6)	*Methylocystis*	*hirsuta*, *rosea*, *echinoides*	4, KRF, Kb, KM, Kerala mud	10^–6^–10^–8^
(7)	***Methylosinus***	***sporium***, *trichosporium*	8 **(1)**, KRF, Kerala mud	10^–6^–10^–8^

*Total strains from each genus are shown, and the number of putative novel species is indicated in brackets followed by sample number.

*Taxa from which novel strains were isolated are marked in bold.*

### Isolation of *Methylomonas, Methylosinus, Methylocystis, Methylomicrobium, Methylomagnum, Methylocaldum*, and *Methylocucumis* Strains

**FIGURE 2:**
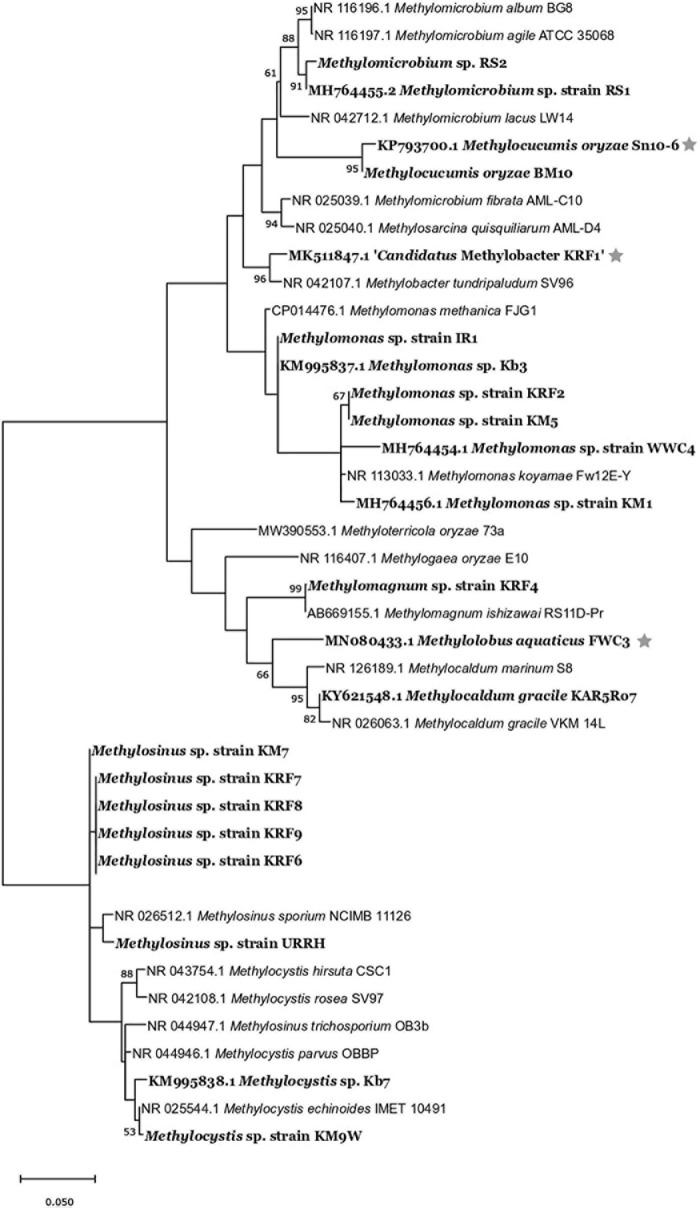
Maximum-likelihood 1,000 bootstrap tree of 16S rRNA-based phylogenetic tree of all the strains (showed in bold) with their closest members. The evolutionary history was inferred by using the maximum-likelihood method and Tamura–Nei model (Tamura and Nei, 1993). Evolutionary analyses were conducted in MEGA X (Kumar et al., 2018). The bar represents 5% divergence. *Strains isolated in our previous studies.

The strains belonged to the following genera: *Methylomonas*, *Methylomicrobium*, *Methylocaldum*, *Methylomagnum*, *Methylocucumis* (Methylococcaceae), and *Methylocystis* and *Methylosinus* (Methylocystaceae) (Table 1 and Supplementary Table 2). Seventeen strains belonging to Methylococcaceae were isolated, and 12 strains belonged to Methylocystaceae. The highest number of strains was from the *Methylomonas* genus (12 strains) followed by the genus *Methylosinus* (eight strains), genus *Methylocystis* (four strains), *Methylomicrobium* (two strains), *Methylomagnum* (one strain), *Methylocucumis* (one strain), and *Methylocaldum* (one strain). *Methylomonas*, *Methylocystis*, and *Methylosinus* were isolated from the majority of samples. The phylogenetic tree (Figure 2) shows that the isolates fall into all the three important clades of proteobacterial methanotrophs. The *Methylomonas* strains majorly showed pink to orange-colored colonies. *Methylomagnum* strain KRF4 represented a new member of *Methylomagnum ishizawai* (Frindte et al., 2017), the first report from India. A *Methylocaldum gracile* strain, KAR5Ro7, was isolated from the Mulshi sample. *Methylocucumis oryzae* strain BM10 represented a new strain of the genus and species *M. oryzae*, thereby being the second strain of this genus and species, which was initially a single species and single strain genus (Pandit and Rahalkar, 2018; Pandit et al., 2018). Thus, Type Ia methanotrophs representatives of *Methylomonas*, *Methylomicrobium*, and *Methylocucumis*; Type Ib *Methylomagnum* and *Methylocaldum*; and Type II *Methylocystis* and *Methylosinus* strains were cultured, covering the three major groups.

### 16S rRNA Gene-Based Next-Generation Sequencing of Three of the Rice Samples

**FIGURE 3:**
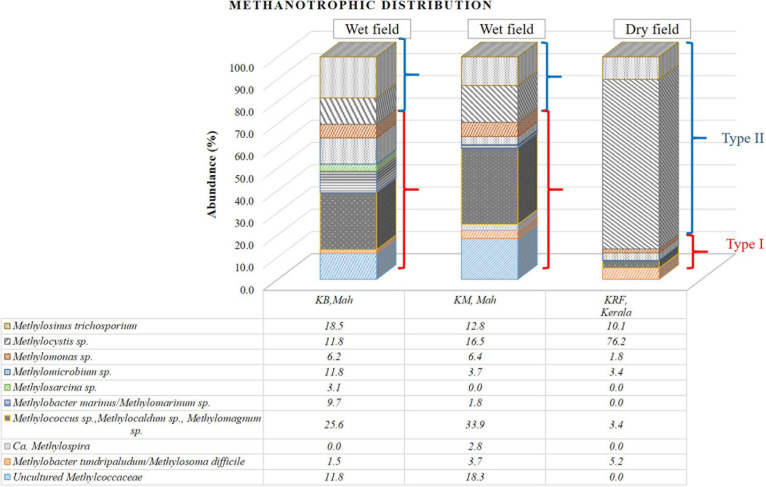
Bar diagram representing the distribution of the sequences obtained from the 16S rRNA gene-based next-generation sequencing (NGS) analysis affiliated to their nearest cultivated members.

The methanotrophic community structure in three rice rhizospheric soils was elucidated using the V3–V4 amplicon NGS in KB and KM samples from the Pune and Konkan regions and KRF sample from the Kerala region to analyze the microbial diversity. The bar diagram (Figure 3 and Table 2) shows the distribution of the sequences.

**TABLE 2:** Total number of sequences of methanotrophs from 16S rRNA gene-based next-generation sequencing (NGS) analysis data.

**Species or lineage**	**Sequences Kb (wet field—rice growing)**	**Sequences KM (rice growing wet)**	**Sequences KRF (winter dry field)**
Methylococcaceae	136	74	58
Methylocystaceae	59	32	385
Ratio of Methylococcaceae/Methylocystaceae	2.3	2.3	0.20

In two of the three samples, KB and KM, a dominance of Methylococcaceae (Type I methanotrophs) was found over Methylocystaceae sequences (Type II methanotrophs). The ratio of Methylococcaceae:Methylocystaceae was ∼2.3 in cases (Table 2). A geographic distance of 117 km separates both these fields. The diversity of Methylococcaceae-related sequences was abundant in both locations, where at least five to six genera were detected within the family Methylococcaceae. Among the abundant genera, the Type Ib group consisting of *Methylolobus* sp., *Methylococcus* sp., *Methylocaldum* sp., and *Methylomagnum* sp. (25.6% and 33.9%) occupied the major abundance in KB and KM samples. Within the Methylococcaceae, members of at least six to seven different genera were detected in each sample (Figure 3). Additionally, the sequences related to Type Ia methanotrophs, *Methylomicrobium* (11.8 and 3.7%), *Methylobacter marinus*/*Methylomarinum* (9.7 and 1.8%), and *Methylomonas* (6.1 and 7.4%) were found in dominance. A minority percentage of 1.5 and 3.7% were occupied by the species *Methylobacter tundripaludum* or *Methylosoma difficile* in samples KB and KM, respectively. In the KM sample, *Methylolobus* sequences dominated and were in equal numbers, and the *Methylocystis*-related sequences were present. Within the family Methylocystaceae (Type II), the genera’s abundance varied in KB and KM samples. Genus *Methylosinus* was abundant in the KB sample with 18.5% followed by 11.8% of genus *Methylocystis*. In the KM sample, the genus *Methylocystis* was abundant with 16.5%, and genus *Methylosinus* occupied 12.8% of the abundance. The third field, KRF, from Kerala, showed a higher number of Methylocystaceae sequences (87%), and the ratio of Methylococcaceae to Methylocystaceae was 0.15 (Table 2). The raw data obtained from the 16S rRNA gene-based NGS of the rice samples have been deposited as PRJNA505747. The phylogenetic tree of the clones from this study belonging to the Type I methanotrophs (Methylococcaceae family) showed major clades close to the genera *Methylomicrobium*, *Methylocaldum*, and *Methylolobus* (Supplementary Figure 1). The phylogenetic analysis of the Type II methanotrophs showed affiliation to *Methylocystis* and *Methylosinus* species (Supplementary Figure 2).

### A Putative New Species of *Methylomicrobium* Strain RS1

A strain RS1 was isolated from a rice stem sample (soil attached to the stem just above the surface), which showed ∼98% 16S rRNA gene similarity (MH764455.1, 1,363-bp sequence) with the 16S rRNA gene of *Methylomicrobium album* ATCC 35068^T^. The partial *pmoA* sequence (∼500 bp) showed 93.9% *pmoA* similarity with the *pmoA* gene of the same organism, *M. album* ATCC 35068^T^. The colonies of RS1 were white to cream in color, and the cells show a fat rod-elliptical cell type of morphology (Supplementary Figure 3). Another strain, RS2, virtually identical and showing 99.9% similarity in the 16S rRNA gene, was also isolated from the seven enriched strains. We further analyzed only RS1 in detail; both of them were nearly identical.

**FIGURE 4:**
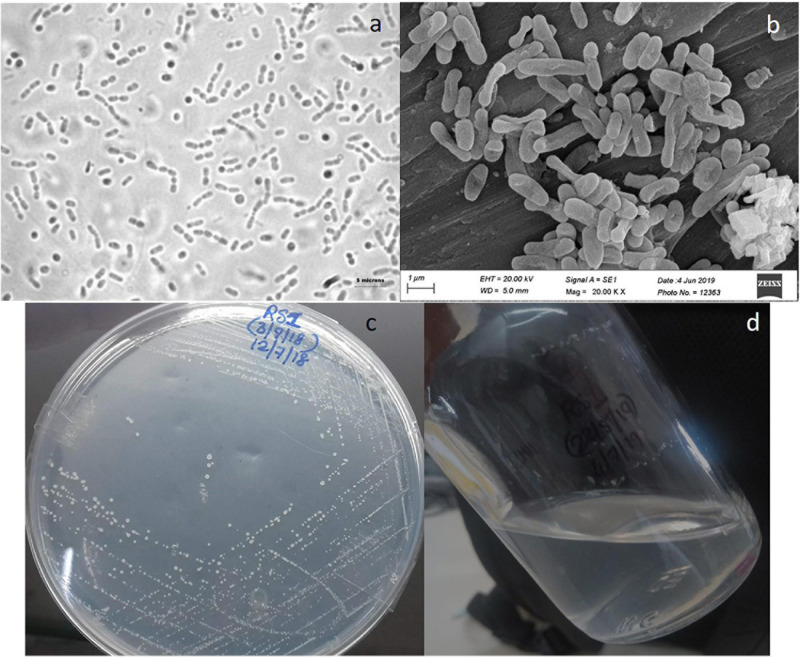
Morphology of strain RS1: **(a)** live cells were observed under a phase-contrast microscope (Nikon 80i, Japan microscope with a camera) under 100× magnification with oil emulsion. **(b)** Culture observed under a scanning electron microscopy (SEM) (Zeiss model EVO-MA-15 SEM). **(c)** Colony morphology on solid nitrate mineral salts (NMS) medium. **(d)** RS1 forms whitish turbid suspension growth in liquid NMS medium.

The morphological details of the strain RS1 are shown in Figure 4. Strain RS1 cells are motile pleomorphic bacilli with the dimensions of 1.0–4.0 μm × 0.5–1 μm (Figures 4A,B). Colonies of RS1 were white, circular, and butyrous in consistency (Figure 4C). The cells have Gram-negative character. The growth of strain RS1 in liquid medium was in the form of white turbidity that attained an OD of 0.15–0.25 (Figure 4D).

After the complete 16S rRNA gene (1,523 bp) was obtained using the sequenced draft genome, the 16S rRNA strain RS1 expressed the closest similarity to 98.69% with *Methylomicrobium agile* ATCC 3508^T^. The complete 16S rRNA gene sequence (1,523 bp) has been submitted to the NCBI database with the accession number MH764455.2. The 16S rRNA gene phylogenetic tree (Supplementary Figure 4A) of strain RS1 and related species showed a distinct phylogenetic position of strain RS1 from its closest type species *M. agile* strain ATCC 35068^T^. Considering the reclassification suggested (Orata et al., 2018), *Methylosarcina lacus*, the next closest affiliate to strain RS1 has been renamed to *Methylomicrobium lacus*, thus reassuring that RS1 belongs to the genus *Methylomicrobium*.

The RS1 *pmoA* gene sequence expressed 94.01% similarity to its closest type species *M. album* strain ATCC 3303^T^, reflected in the phylogenetic analysis (Supplementary Figure 4B).

### Whole Draft Genome Analysis of RS1

The whole genome of RS1 is 4.73 Mbp in size with a G+C content of 55%. The NCBI accession number for the whole-genome sequence and the bio-project numbers are JAERVK01 and PRJNA690655, respectively. The general draft genome characteristics of strain RS1 are listed in Table 3. The ANIb, AAI, and DDH values between RS1 and its closest members genomes are 69–91, 62–90, and 18–44%, respectively (Supplementary Table 3), which are lesser than the thresholds for prokaryotic species description (95% for ANI and AAI while 70% for DDH) (Kim et al., 2014). Additionally, the 98.69% similarity based on the complete 16SrRNA gene with the type species *M. album* strain ATCC 35068^T^ is also lower than the 98.7% cutoff mentioned for a novel species (Sangal et al., 2016). Based on this analysis, we propose a new name for the putative novel species as ‘*Ca. Methylomicrobium oryzae*’ strain RS1. *M. oryzae* sp. nov. (o.ry’zae. N.L. gen. n. *oryzae*), of rice, referring to isolation from rice plant.

**TABLE 3:** Genomic features of *Methylomicrobium* RS1.

**Traits**	**Values**
Genome size	4.73 Mbp
G + C content	55%
N50	233,876
Proteins (according to NCBI)	4,114
Genes (according to NCBI)	4,231
tRNA, ncRNA	45,4
rRNA	3
Contigs	69
Bioproject number	PRJNA690655
Sequencing platform used	Illumina HiSeq 2 × 150
NCBI accession number	JAERVK0.1

## Discussion

### Highlights of the Method Including Advantages and Disadvantages Over the Traditional Method of Enrichment and Isolation of Methanotrophs

Due to the various challenges, the cultivation of methanotrophs has been attempted by very few research groups in the world. The number of cultivated methanotrophs is far less than the as-yet-uncultivated ones (Hamilton et al., 2015). Despite all the knowledge regarding methanotroph cultivation, the isolation of novel methanotrophic taxa remains a challenging task (Dedysh and Dunfield, 2014).

In our approach, we used a medium (modified dil. NMS) that was modified from the dilute NMS medium for culturing of lake methanotrophs (Bussmann et al., 2004; Rahalkar et al., 2007; Deutzmann et al., 2014; Schink and Rahalkar, 2016). The dil. NMS medium was a modification of the original NMS medium described by Whittenbury et al. (1970). We modified the dil. NMS medium used for lake methanotrophs to cultivate rice field methanotrophs by increasing the amount of KNO_3_ to 0.25 g/L (Pandit et al., 2016). The use of five times more nitrate (KNO_3_) concentration in the modified dNMS medium as compared with the original one (Bussmann et al., 2004) was because rice field habitats are fertilized with nitrogenous fertilizers like urea or ammonium phosphates. Our group has used this medium in successfully isolating representatives of two novel Type I methanotroph genera (Pandit et al., 2018; Pandit and Rahalkar, 2019; Rahalkar et al., 2020) and new species (Rahalkar and Pandit, 2018; Khatri et al., 2019; Rahalkar et al., 2019) from Indian rice fields and wetland environments.

Additionally, we used serial dilutions, initially in serum bottles, followed by a second dilution series in microtiter plates. The first serial dilution culturing would give a chance to numerically dominant but slow-growing methanotrophs due to a long incubation of 6–8 weeks. In contrast, in traditional culturing, usually a single enrichment is set up, or dilutions are directly plated on plates (Dedysh and Dunfield, 2014). The single-enrichment series would allow only the growth of the fast-growing methanotrophs. Moreover, many methanotrophs are resistant to culturing on solid agar plates directly. The purpose of using a second dilution series was a separation of the diverse methanotrophs growing in the first dilution series bottles. The advantage of using microtiter plates for the enrichment of methanotrophs has been documented earlier (Bussmann et al., 2004; Bowman, 2006).

The first step of enrichment was from 10^–1^ to 10^–10^, followed by a second dilution series of up to 10^–8^. Preincubated rice field soils favored the cultivable methanotrophs to range from 10^7^ to 10^9^ (Escoffier et al., 1997). Most probable number (MPN)-based studies on the rice rhizosphere soils of Uruguay and India revealed the methane-oxidizing bacteria (MOB) counts per gram of dry soil to be in the range 1.7 × 10^4^ to 3.3 × 10^4^ (Ferrando and Tarlera, 2009) and 5.5 × 10^8^ cells (Dubey et al., 2003). In a study on the Italian rice field, the count of the methanotroph quantified with real-time PCR revealed that count was in the range of 2.5 × 10^6^ MOB per gram of fresh soil (Kolb et al., 2003). In our study, we have reported successful cultivation of methanotrophs from 10^–2^ to 10^–8^ dilutions, thus representing the MOB count to be in this range.

In the final step, we used the same medium with agarose for the separation of isolates and repeated the streaking and re-streaking. Methanotrophs are usually associated with other heterotrophic bacteria (Dedysh and Dunfield, 2014; Knief, 2015; Kalyuzhnaya et al., 2019). The heterotrophs sometimes thrive on the media components like agar, or the metabolites secreted by the methanotrophs like acetate, formate, lactate, and succinate (Bothe et al., 2002; Kalyuzhnaya et al., 2013); methanol (Wilkinson et al., 1974); formaldehyde (Bussmann et al., 2006); and exopolysaccharides (Koch et al., 2008; Dedysh et al., 2012). These heterotrophs form satellite colonies around the methanotrophic ones on solid growth media. The most challenging, laborious, and time-consuming task in methanotroph isolation is removing these satellite bacteria (Dedysh and Dunfield, 2014). Numerous methanotrophs cannot grow or poorly grow on agar (Bowman, 2006); therefore, a purified form of agar, i.e., agarose, was used in our prior studies (Rahalkar et al., 2007). Therefore, in our current method, we have used agarose to allow better growth on solid medium.

Although the current discussed method is little time-consuming due to long incubation periods as compared with the traditional way (Dedysh and Dunfield, 2014), a relatively large number of methanotroph strains from various genera were isolated using the current method. For example, a total of 21 methanotrophs from four different genera were isolated from the Kerala rice field samples, which included including a putative novel species *Ca. M. oryzae* KRF1, described in an earlier publication (Rahalkar et al., 2019; Khatri et al., 2020). In the current study, six methanotroph strains belonged to three genera, and a putative novel species *Ca. Methylomicrobium oryzae* strain RS1 was from the Malegaon region. Cultivation studies on Japanese rice field samples had resulted in the isolation of 13 strains belonging to three different genera (Dianou et al., 2012). Similarly, a culturing experiment on Uruguay rice field methanotroph samples had resulted in culturing of strains from three genera (Ferrando and Tarlera, 2009).

### Comparison of the Cultured Community With Uncultured Methanotroph Members

Rice fields are among the most important anthropogenic sources of atmospheric methane and contribute to 10% of the global methane emissions (Conrad, 2009). India leads the world in the rice production area with ∼43.7 m ha under rice cultivation and covers 28.6% of the world’s rice cultivated area^8^. Indian rice fields emit less methane (3.9 Tg/year) (Ganesan et al., 2017), which is ∼15% of the global emissions from rice fields (25.6 Tg/year) (Yan et al., 2009). Methanotrophs present in rice rhizospheres have been estimated to oxidize about 20% of the produced methane (Conrad, 2009). The microbial ecology of methanotrophic bacteria from Indian rice fields of the Northern region (subtropical) has been studied mainly by culture-independent methods (Vishwakarma et al., 2009; Vishwakarma and Dubey, 2010a, b). However, significantly less is known about the cultural identity of methanotrophs from the tropical rice ecosystems. Cultivated methanotrophs from rice fields belong to the genera: *Methylomonas*, *Methylobacter*, *Methylogaea*, *Methylomagnum*, *Methyloterricola*, *Methylocaldum*, *Methylocucumis*, *Methylocystis*, and *Methylosinus* (Ferrando and Tarlera, 2009; Dianou et al., 2012; Pandit et al., 2016; Pandit and Rahalkar, 2018; Pandit et al., 2018; Khatri et al., 2019; Rahalkar et al., 2019). Growth curves of selected strains indicated that maximum growth of up to 0.15–0.2 OD was achieved within 15 days. A recent carbon and nitrogen source optimization study using representative strains from *Methylocystis*, *Methylosinus*, *Methylomicrobium*, and *Methylomonas* showed a similar range growth when methane was used (Tays et al., 2018) (OD value increased up to 0.2). In order to use the strains for biotechnological purposes, it would be necessary to optimize the medium and conditions for optimal growth.

The sampling was done in the 80–90 days’ period after transplantation in most of the cases, which marks the late flowering or grain formation stage. The highest methane oxidation activity has been reported at this stage from Indian soils (Vishwakarma et al., 2009). In the present study, among the cultivated species, the maximum species belonged to the genus *Methylomonas*, followed by *Methylocystis* or *Methylosinus*, as reported in a study from the Japanese rice fields (Dianou et al., 2012). Also, we report the cultivation of strains of *M. ishizawai* and *M. oryzae*, which have been typically isolated from rice fields (Khalifa et al., 2015; Pandit et al., 2018; Pandit and Rahalkar, 2019). Strain KRF4 (Supplementary Figure 3) showed characteristic fat oval-cylindrical cells, which were large-sized, identical to strains 114 and 175, isolated from the Philippines (Frindte et al., 2017).

We supported our study with 16S rRNA gene-based NGS of three representative rice rhizosphere soils. The major players detected in the field KB were *Methylomicrobium* and *Methylobacter* from Type Ia methanotrophs, and *Methylolobus* and *Methylomagnum* (a new genus in the description) from Type Ib and Type II methanotrophs (*Methylocystis* and *Methylosinus*). The rice field KM showed the dominance of mainly Type Ib (*Methylolobus* and *Methylomagnum*) and Type II methanotrophs (*Methylocystis* and *Methylosinus*). KRF field showed the dominance of Methylocystaceae family, which could be due to the time of sampling, as the KRF sample was taken in December (December 2017) where the rice fields were dry, whereas the other two samples were taken in peak monsoon when the rice fields were completely waterlogged. In rice fields that undergo drying, it has been reported that Methylocystaceae forms a more abundant and constant population, whereas Methylococcaceae members are dynamic and are more diverse and active (Vishwakarma et al., 2009; Ma et al., 2013; Lüke et al., 2014; Leng et al., 2015 and references therein).

Comparison of the cultivated members with the members detected by metagenomics revealed that most of the dominant methanotrophic lineages detected in rice fields were cultivated. Together with the current cultivation study and our previous studies, we have broadened the culturable diversity of methanotrophs from Indian tropical rice fields by culturing from total of nine genera of methanotrophs.

The methanotrophic community detected in Indian rice field samples (KRF, KM, and KB) was very similar to the methanotrophs seen in Vercelli, Italy. In a stable isotope study, the community was composed of *Methylomonas*, *Methylobacter*, *Methylomicrobium*, *Methylocaldum*, *Methylococcus*, *Methylocystis*, and *Methylosinus* (Shrestha et al., 2008). Similarly, in China, a very similar methanotroph community has been detected (Ma et al., 2013). The distribution of Methylococcaceae to Methylocystaceae in various tropical and subtropical regions has been found to be variable, and this was recently studied using *pmoA* amplicon sequencing (Lüke et al., 2014). In this work, they compared the methanotroph population in tropical paddy fields distributed through the two main islands of Indonesia (Java and Sumatra) and from South Southern Vietnam (Tien Giang province) to the wetland rice fields located in the subtropical climate: the coastal area south of the Yangtze River Delta (Cixi, Zhejiang Province, China) and Vercelli Province, Italy (Lüke et al., 2014). The tropical wetlands showed the dominance of Methylococcaceae, in general (Type Ia and Type Ib together), whereas in the subtropical area, Type II methanotrophs were dominant. This was similar to our results where we found a dominance of Methylococcaceae (Types Ia and Ib) over Methylocystaceae in both the fields, which fall under the tropical regime. Our earlier documented study also showed the dominance of Methylococcaceae from Maharashtra rice fields (Pandit et al., 2016). At the same time, the studies from Northern parts of India (Varanasi) have documented the dominance of Methylocystaceae members (Vishwakarma et al., 2009) with subtropical weather. Also, type of soil and cultivation practices have been shown to impact the methanotroph community structure, as indicated by studies from Northern India (Vishwakarma et al., 2009; Vishwakarma and Dubey, 2010b). Hence, the differences in the community structure of methanotrophs observed could be due to several reasons stated above.

### Cultivation of Yet Uncultivated Taxa (*Methylomicrobium*)

Despite indications of *Methylomicrobium* from the rice ecosystems studied worldwide, there had been no previous strains of *Methylomicrobium* isolated reported from rice field habitats. A recent study reported the presence of methanotrophic taxa distantly related to genus *Methylomicrobium*, *Methylosarcina*, *Methylocaldum*, and *Methylocystis* in six rice paddies studied across Taiwan (Shiau et al., 2018). The methane consumption was dominated by the *Methylomicrobium*/*Methylosarcina* like methanotrophs in the forest soils of Vercelli, Italy, as observed using the stable isotope probing–phospholipid fatty acid (SIP-PLFA) profiling method (Mohanty et al., 2006). In yet another study of urea fertilized rice field from Vercelli, Italy, using the RNA-SIP methodology, the uptake of ^13^CH_4_ was exclusively observed in the genera *Methylomicrobium* and *Methylocaldum* (Noll et al., 2008). The reported *Methylomicrobium* species, *M. agile* (Bowman et al., 1995), *M. album* (Bowman et al., 1993), *Methylomicrobium alcaliphilum* (Kalyuzhnaya et al., 2008), *Methylomicrobium buryatense* (Kaluzhnaya et al., 2001), *Methylomicrobium japanense* (Kalyuzhnaya et al., 2008), *Methylomicrobium kenyense* (Kalyuzhnaya et al., 2008), *M. lacus* (Kalyuzhnaya et al., 2005; Shrestha et al., 2008; Oren and Garrity, 2019), and *Methylomicrobium pelagicum* have been isolated from niches like freshwater swamp sediment, soda lake sediments, lake sediments, marine sediment, and estuarine sediments/waters. The novel member of *Methylomicrobium*, which we have isolated, could be one of the first members of *Methylomicrobium*, isolated from any rice field in the world.

Methanotrophs are natural methane mitigation agents. The methanotroph isolates isolated from native habitats such as rice fields enable us to use them as models for studying methane mitigation. The successful cultivation of methanotrophs from various natural habitats opens an avenue for various biotechnological applications. Problems associated with cultivation and obtaining fast growth as well as cryopreservation still limits their applications. The following applications have already been reported for methanotrophs. The production of polyhydroxyalkanoate (PHA) granules using *Methylocystis* and *Methylosinus* strains (Karthikeyan et al., 2015), and *Methylomagnum* (Luangthongkam, 2019); methanol using *Methylomicrobium* (Patel et al., 2020a), *Methylosinus* (Patel et al., 2016), *Methylomonas* (Patel et al., 2018b), and *Methylocystis* (Patel et al., 2018a); and carotenoids using *Methylomonas* (Ye et al., 2007) etc. can be analyzed. In addition to these applications, other applications such as bioremediation, biosensor preparation, denitrification, microbial fuel cells, oxidation of alkanes and aromatic compounds, and utilization of CO_2_ (Chistoserdova, 2018) can also be explored.

In conclusion, our method resulted in the isolation and cultivation of uncultured, dominant, and environmentally significant methanotrophs from Indian rice fields. The cultivation strategy where we used a low-nutrient medium, two series of serial dilutions, and long-term incubations could have been an essential strategy for the isolation. We cultivated methanotrophs from rice field soils from India using a culturing method with a double-step dilution till extinction cultivation followed by isolation on solid plates. The ∼29 strains belonged to seven genera: *Methylomonas* (18), *Methylocaldum* (one), *Methylomicrobium* (one), *Methylomagnum* (one), and *Methylocucumis* (one) from Type I methanotrophs (Methylococcaceae), and *Methylocystis* (three) and *Methylosinus* (five) from Type II methanotrophs (Methylocystaceae). Culture-independent molecular analyses using the V3–V4 amplicon sequencing showed that methanotrophs from nine or 10 genera were present in rice rhizosphere samples. The cultured putative novel member of *Methylomicrobium* represents the first cultured *Methylomicrobium*, detected in rice fields. The isolates obtained in this study would be used as model or reference strains to study methanotrophy in rice fields and could be used for further environmental and biotechnological applications.

## Data Availability Statement

The datasets generated for this study can be found in online repositories. The names of the repository/repositories and accession number(s) can be found in the article/Supplementary Material.

## Author Contributions

MR designed the experiments, collected the samples, guided all the experiments, did the sequence analysis, 16S rRNA gene-based NGS analysis, phylogenetic analysis, assisted in the figures, and wrote the manuscript. PP and KK performed the enrichment, isolation of the organisms, subculturing and maintenance of cultures, DNA extraction, and sample preparation for sequencing and some part of the blast analysis. JM did the subculturing of cultures and helped in DNA extraction, etc. RB did the sampling, performed 16S rRNA gene-based NGS analysis and phylogenetic analysis, and prepared the figures for phylogenetic trees. All authors reviewed the manuscript and approved the final version.

## Conflict of Interest

The authors declare that the research was conducted in the absence of any commercial or financial relationships that could be construed as a potential conflict of interest.

## Publisher’s Note

All claims expressed in this article are solely those of the authors and do not necessarily represent those of their affiliated organizations, or those of the publisher, the editors and the reviewers. Any product that may be evaluated in this article, or claim that may be made by its manufacturer, is not guaranteed or endorsed by the publisher.

## Footnotes

1
http://rast.nmpdr.org/

2
https://www.ncbi.nlm.nih.gov/genome/annotation_prok/

3
http://jspecies.ribohost.com/jspeciesws/#analyse

4
http://ggdc.dsmz.de/

5
http://enve-omics.ce.gatech.edu/aai/

6
https://mafft.cbrc.jp/alignment/server/

7
https://www.ncbi.nlm.nih.gov/

8
https://www.statista.com/statistics/765691/india-area-of-cultivation-for-rice/

## Supplementary Material

Supplementary Figure 1 Maximum-likelihood bootstrap tree of the 16S rRNA gene sequences of *Methylococcaceae* methanotrophic isolates in comparison with those of other clones obtained from KB and KM rice rhizosphere samples. The number in front of each denotes the number of sequences from that sample, e.g., KB15 (15 partial 16S rRNA gene sequences from KB sample). The evolutionary analyses were conducted in MEGA7. The bar represents 1% divergence. The tree was drawn to scale, with branch lengths in the same units as those of the evolutionary distances used to infer the phylogenetic tree. The evolutionary distances were computed using the Maximum Composite Likelihood method and are in the units of the number of base substitutions per site. The analysis involved 271 nucleotide sequences. All positions containing gaps and missing data were eliminated. There were a total of 134 positions in the final dataset.

Supplementary Figure 2 *Methylocystaceae* family phylogenetic tree of the V3–V4 sequences derived from the 16S rRNA gene-based next generation sequencing (NGS) analysis using the KB and the KM samples. The number against the sample name represents the number of sequences retrieved for that sample. The tree also consists of methanotrophic isolate sequences for co-relation with the sequences. The phylogenetic tree was constructed using MEGAX, and the bar represents 1% divergence. The analysis involved 104 nucleotide sequences. All positions containing gaps and missing data were eliminated.

Supplementary Figure 3 Growth of isolates on solid media plates and their respective phase-contrast images with 1,000× magnification. The bar represents 5 μm.

Supplementary Figure 4 **(A)** Maximum-likelihood 1,000 bootstrap tree of the 16S rRNA gene sequence of strain RS1 in comparison with the closely associated *Methylococcaceae* bacteria. The phylogenetic tree was constructed using MEGA X and analyzed based on the Tamura–Nei model. Bar represented 1% divergence. **(B)** Maximum-likelihood 1,000 bootstrap tree of the partial *pmo*A nucleotide sequence of strain RS1 in comparison with the *pmo*A gene sequences of closely related Type I methanotrophs. The evolutionary history was inferred by using the maximum-likelihood method based on the Tamura-Nei model with MEGA X. The bar representing a 5% divergence.

Supplementary Material Growth curves and methane oxidation of the cultures.
