# Supplementary material for: Cultivation of Important Methanotrophs From Indian Rice Fields
Source: Front Microbiol. 2021 Sep 3;12:669244. doi: 10.3389/fmicb.2021.669244 (PMC8447245; doi:10.3389/fmicb.2021.669244)
Supplement: Supplementary Figure 1 — Maximum-likelihood bootstrap tree of the 16S rRNA gene sequences of Methylococcaceae methanotrophic isolates in comparison with those of other clones obtained from KB and KM rice rhizosphere samples. The number in front of each denotes the number of sequences from that sample, e.g., KB15 (15 partial 16S rRNA gene sequences from KB sample). The evolutionary analyses were conducted in MEGA7. The bar represents 1% divergence. The tree was drawn to scale, with branch lengths in the same units as those of the evolutionary distances used to infer the phylogenetic tree. The evolutionary distances were computed using the Maximum Composite Likelihood method and are in the units of the number of base substitutions per site. The analysis involved 271 nucleotide sequences. All positions containing gaps and missing data were eliminated. There were a total of 134 positions in the final dataset. [file Data_Sheet_2.docx]

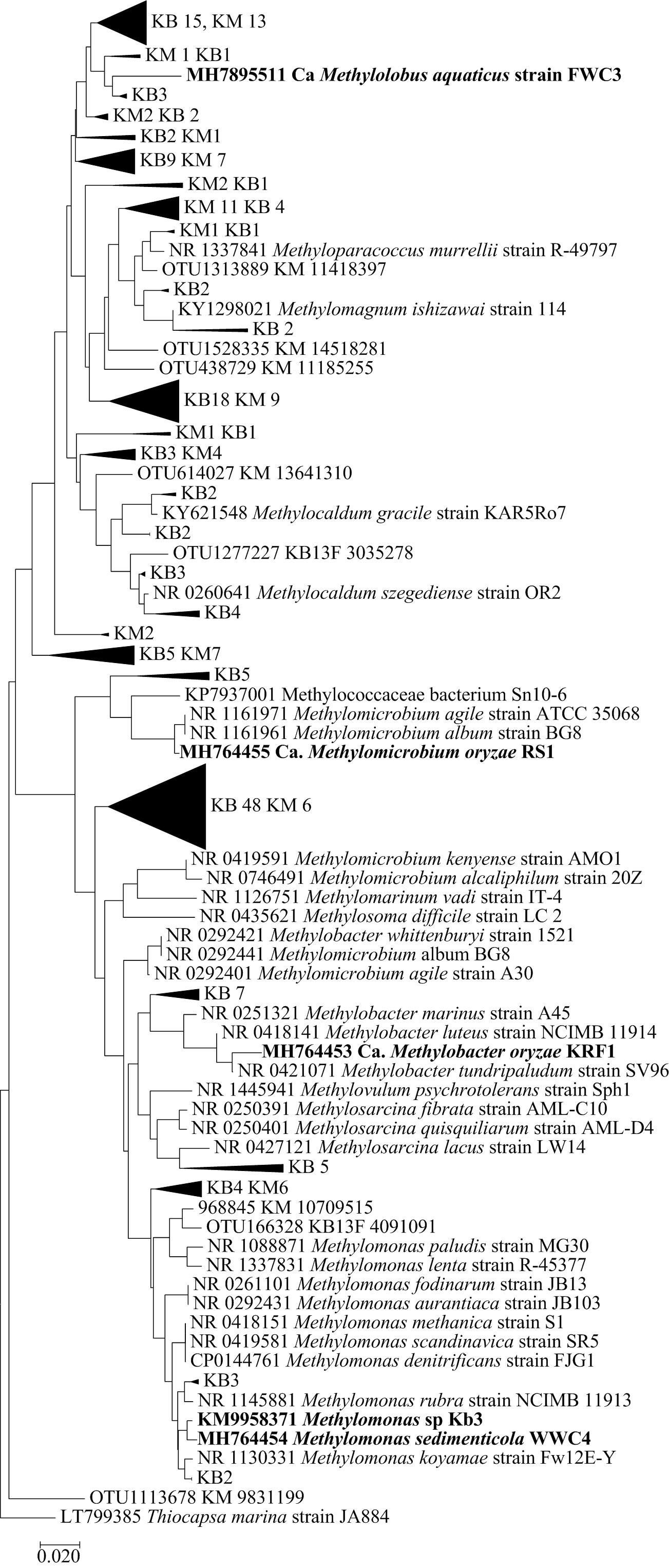


**Supplementary Figure 1:** Maximum-likelihood bootstrap tree of the 16S rRNA gene sequences of *Methylococcaceae* methanotrophic isolates in comparison with those of other metagenome clones obtained from KB (KB in Figure) and KM (KM in Figure) rice rhizosphere samples. The number in front of each denotes the number of metagenome sequences from that sample, e.g., KB 15 (15 metagenome sequences from the KB sample). Evolutionary analyses were conducted in MEGA7. The bar represents 1% divergence


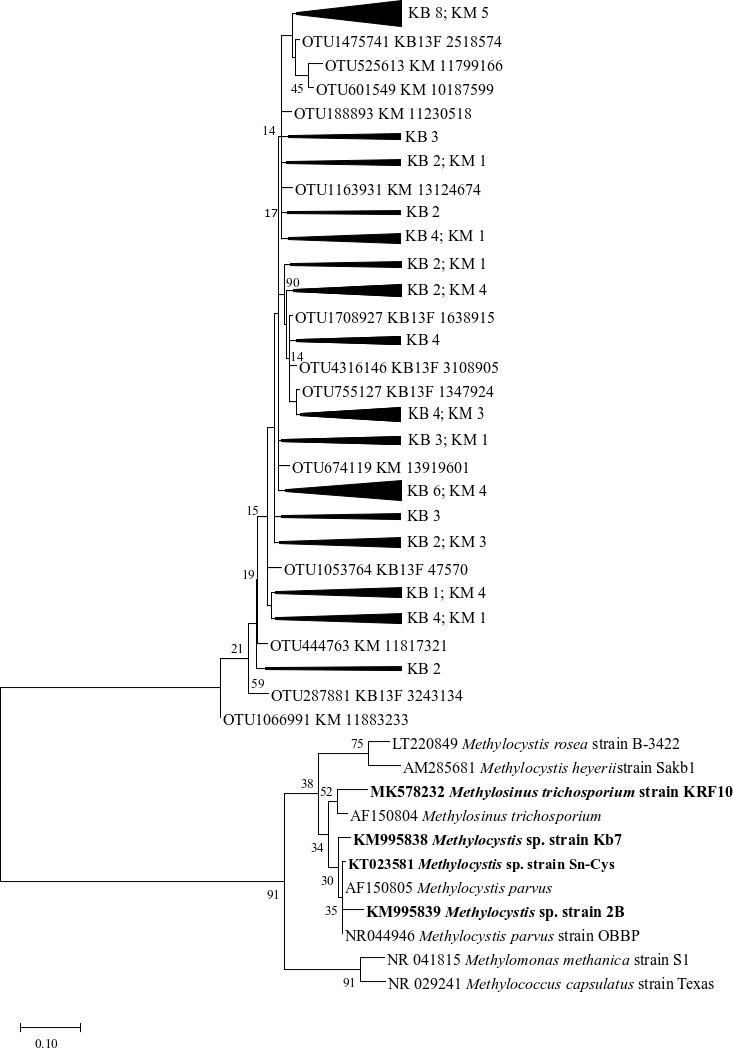


**Supplementary Figure 2:** *Methylocystaceae* family phylogenetic tree of the V3-V4 sequences derived from the metagenome of the KB (KB in the Figure) and the KM (KM in the Figure) samples. The number against the sample name represents the number of sequences retrieved for that sample. The tree also consists of methanotrophic isolate sequences for co-relation with the metagenome sequences. The phylogenetic tree was constructed using MEGAX, and the bar represents 1% divergence

| **Supplementary Figure 3: Growth of isolates on solid media and their respective phase contrast image** | | | | |
| --- | --- | --- | --- | --- |
| **S. No.** | **Sample** | **Strain name** | **Plate image** | **Phase contrast** |
| **1** | Kerala rice field rhizosphere | KRF 2 | 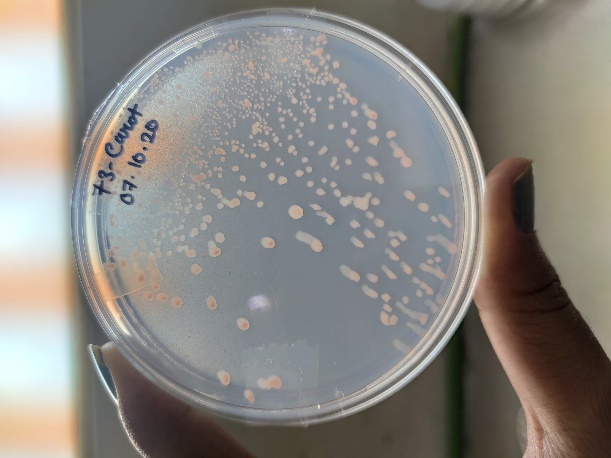 | 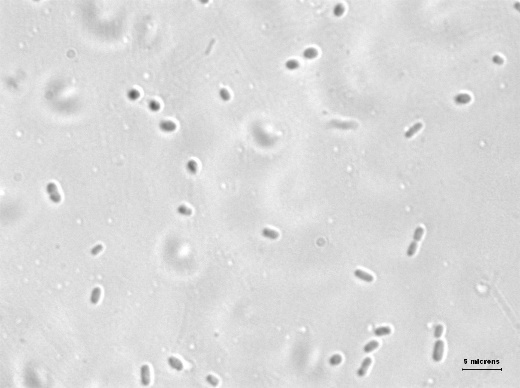 |
| **2** |  | KRF 3 | 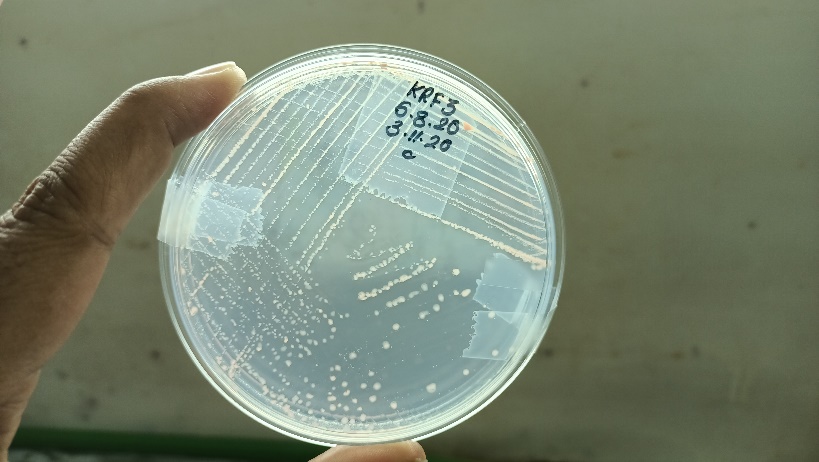 | 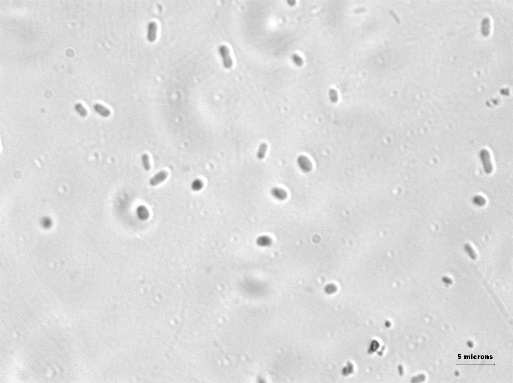 |
| **3** |  | KRF 4 | 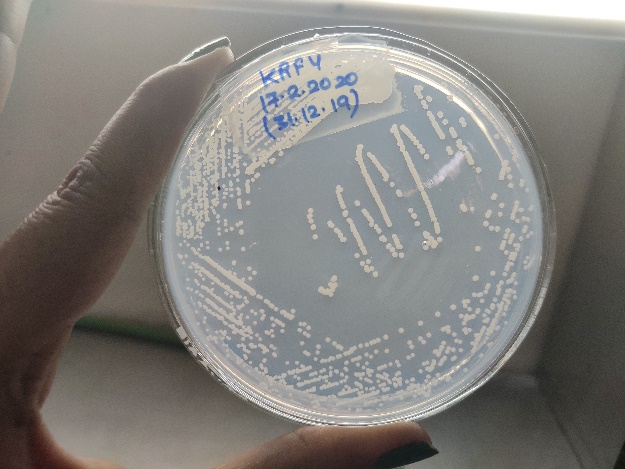 | 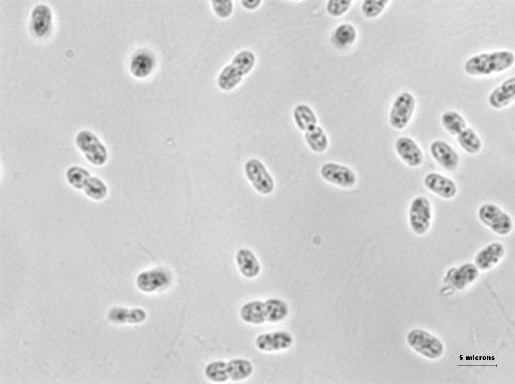 |
| **4** |  | KRF 5 | 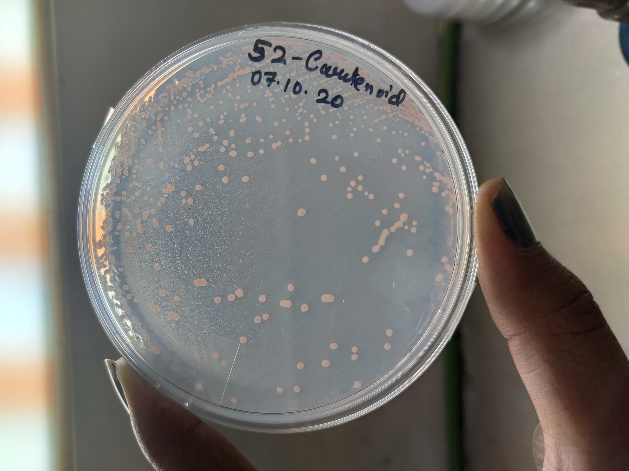 | 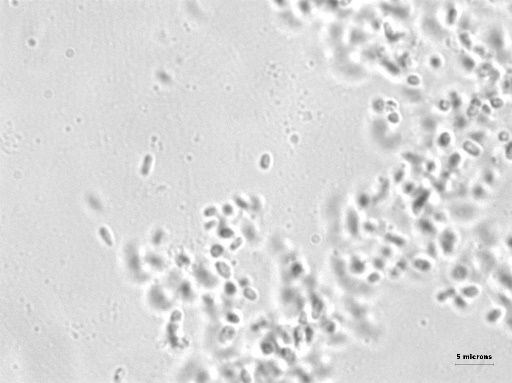 |
| **5** |  | KRF 6 | 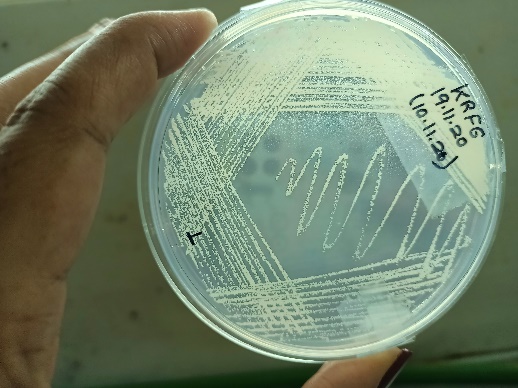 | 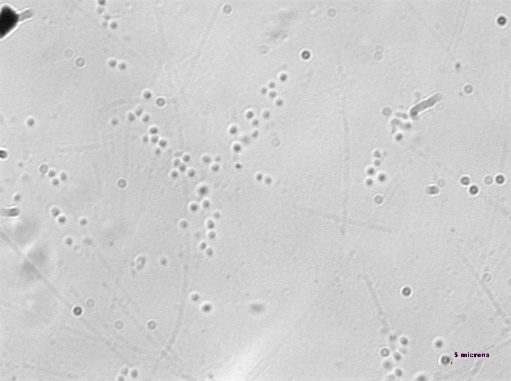 |
| **6** |  | KRF 7 | 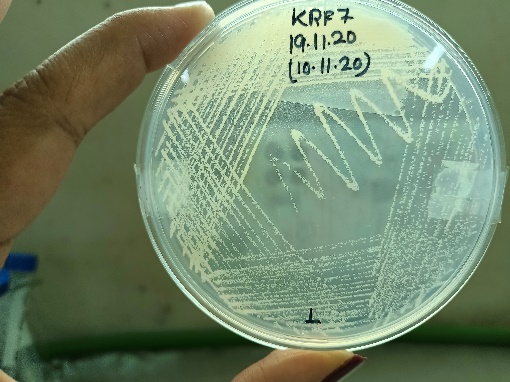 | 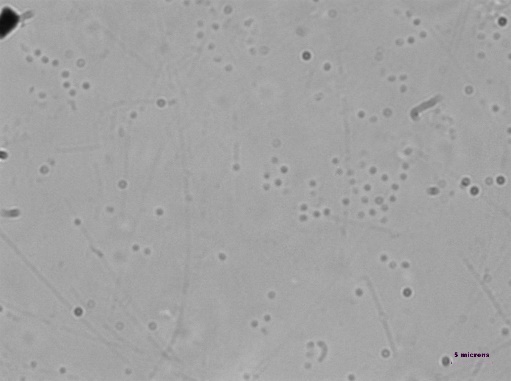 |
| **7** |  | KRF 8 | 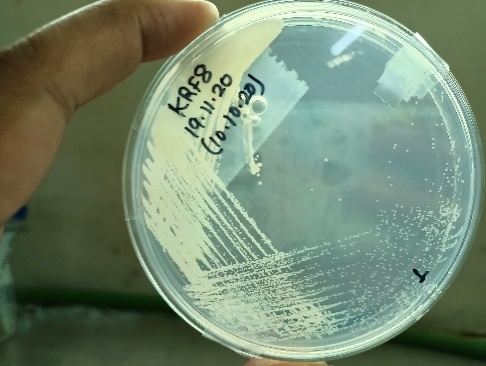 | 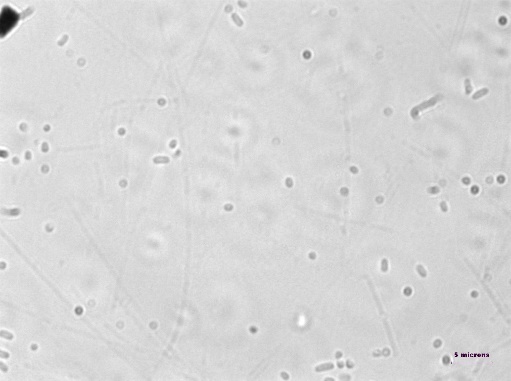 |
| **8** |  | KRF 9 | 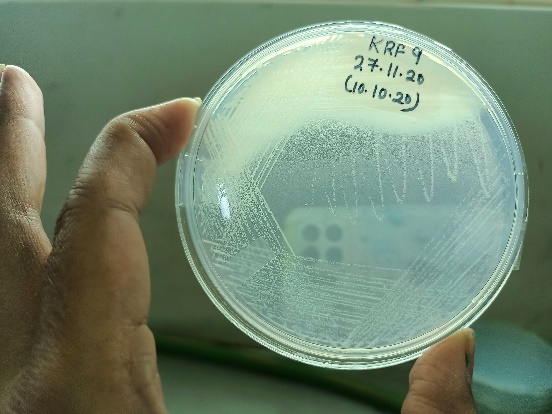 | 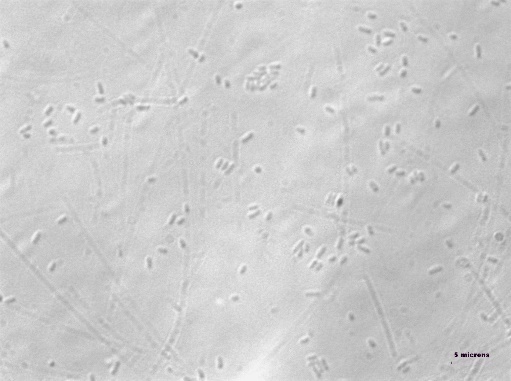 |
| **9** |  | KRF 10 | 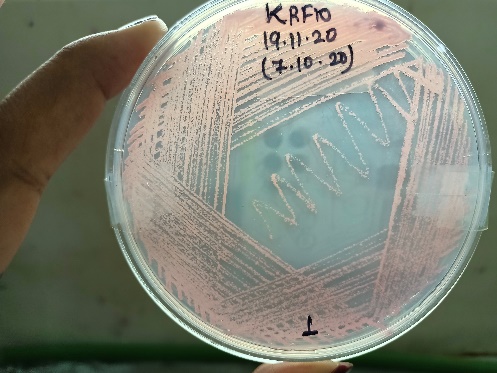 | 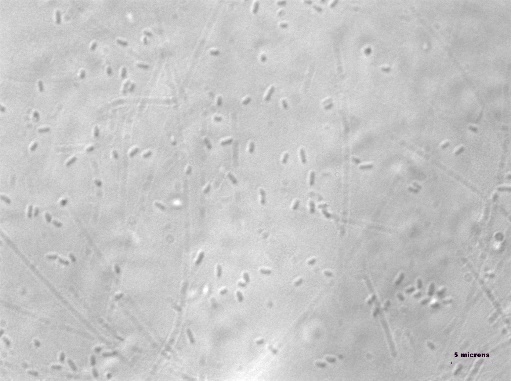 |
| **10** |  | KM 2 | 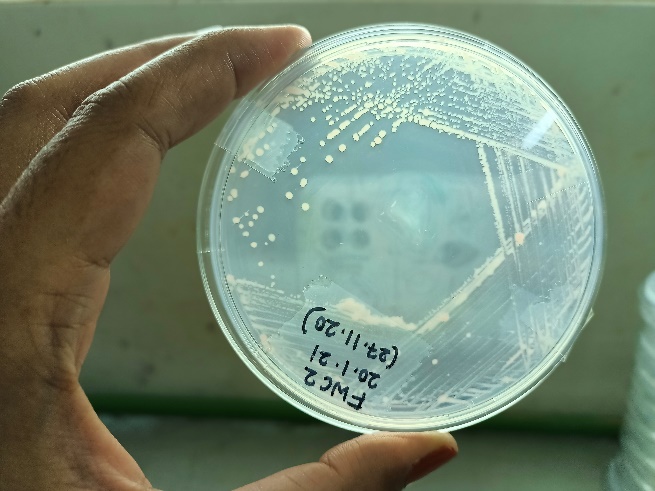 | 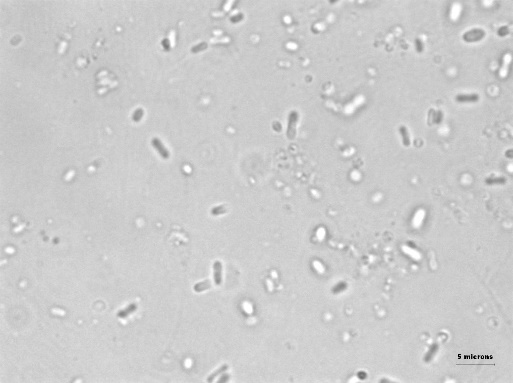 |
| **11** |  | KM 3 | 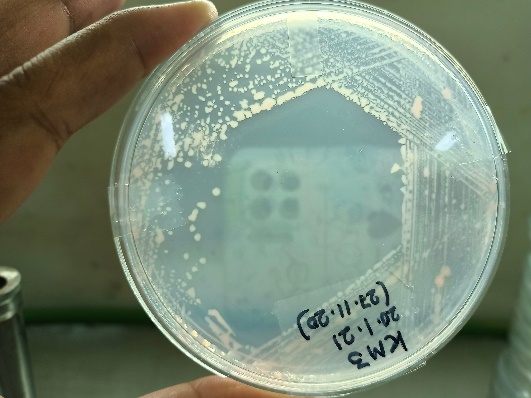 | 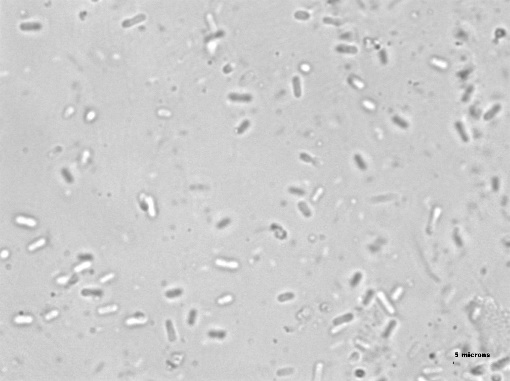 |
| **12** |  | KM 4 | 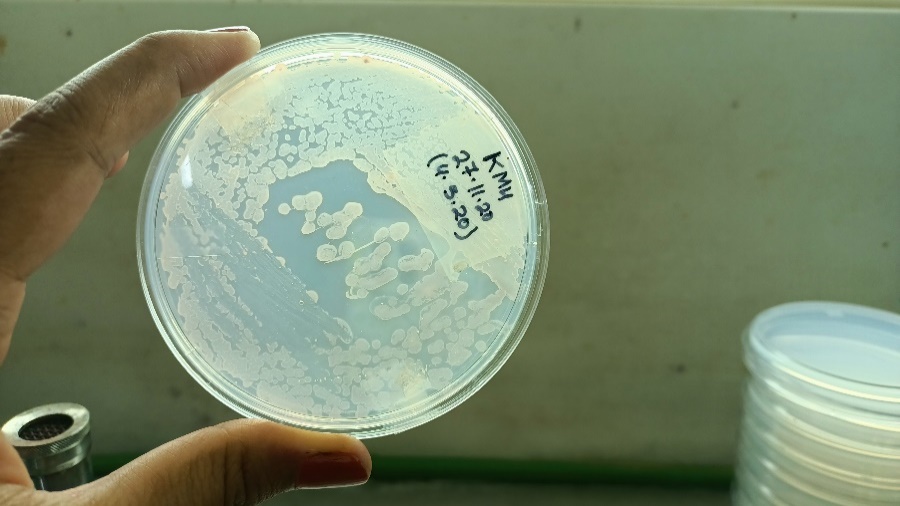 | 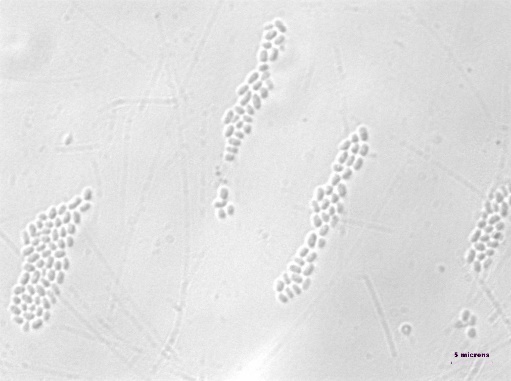 |
| **13** |  | KM 5 | 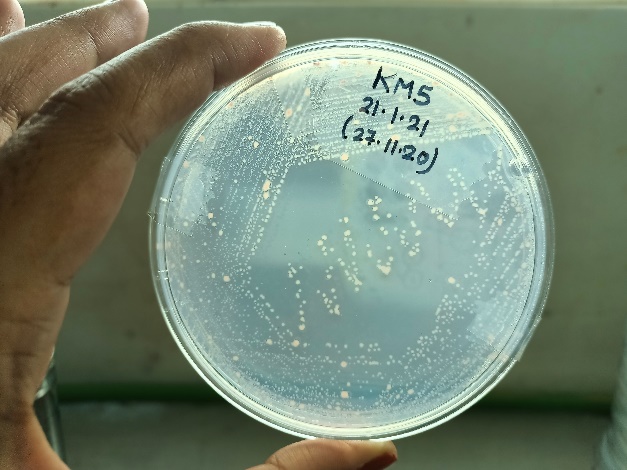 | 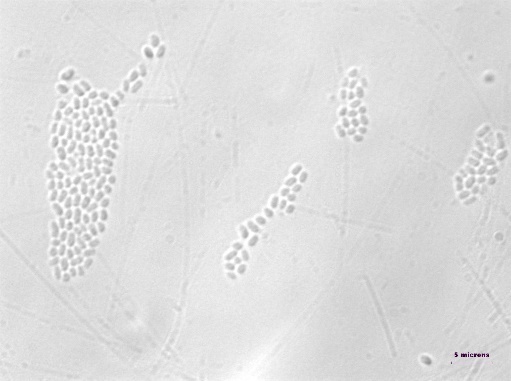 |
| **14** |  | KM 6 | 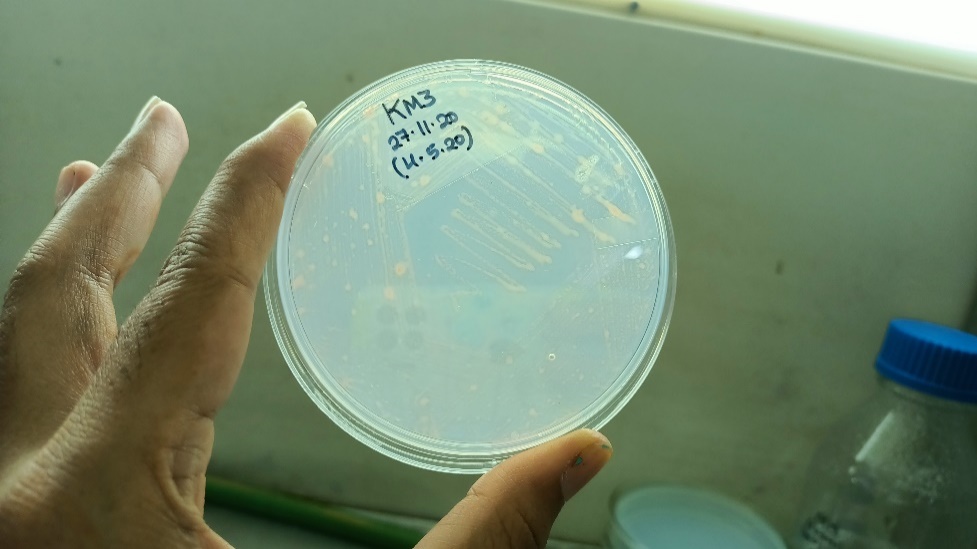 | 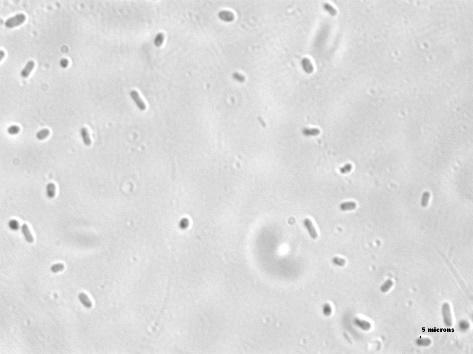 |
| **15** |  | KM 7 | 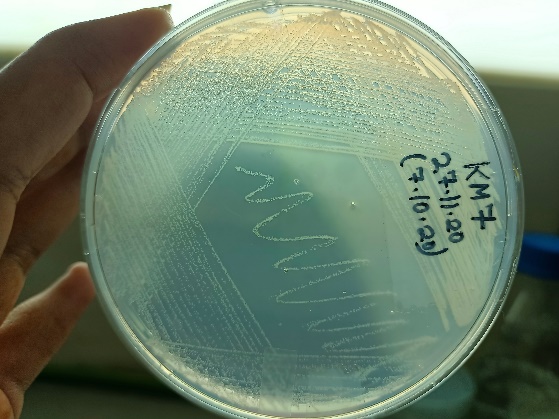 | 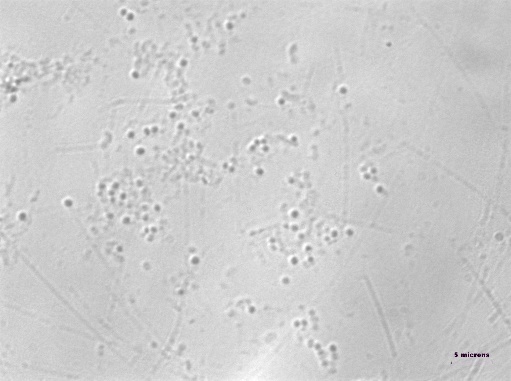 |
| **16** |  | KM 8 | 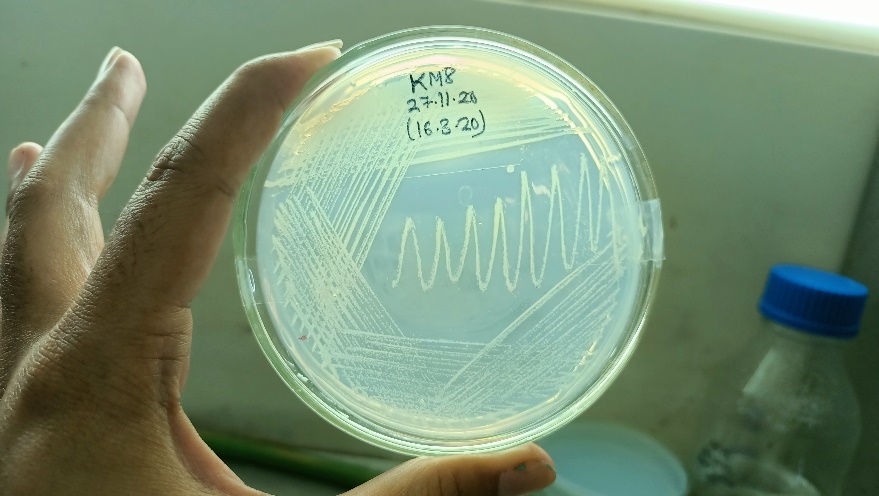 | 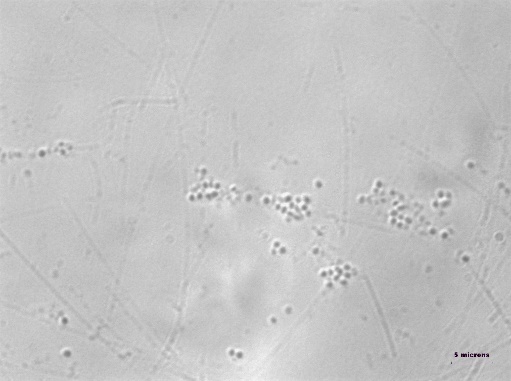 |
| **17** |  | KM 9B | 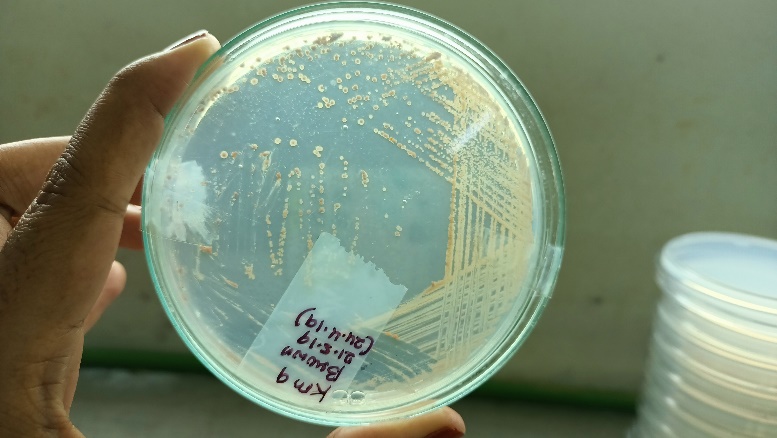 | 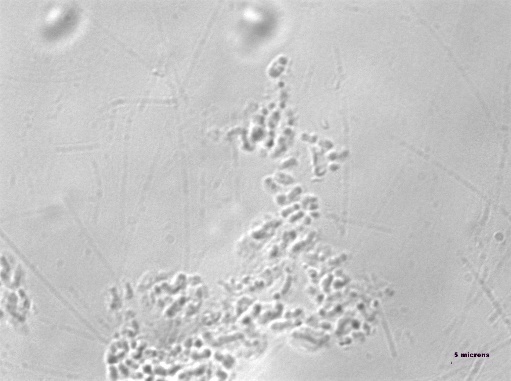 |
| **18** |  | KM 9W | 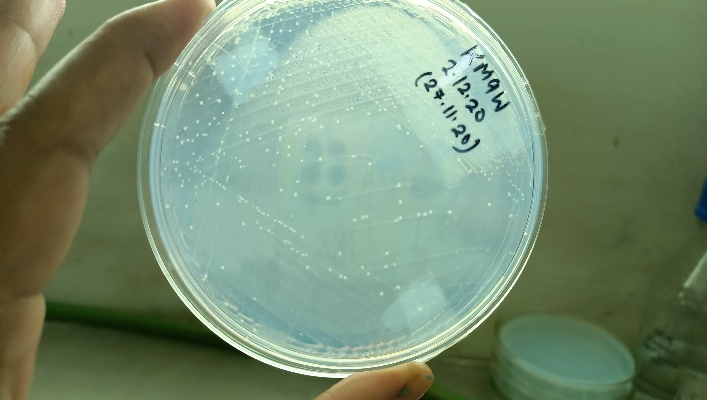 | 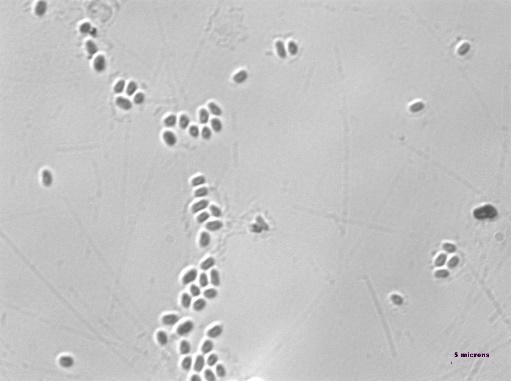 |
| **19** |  | KM 10 | 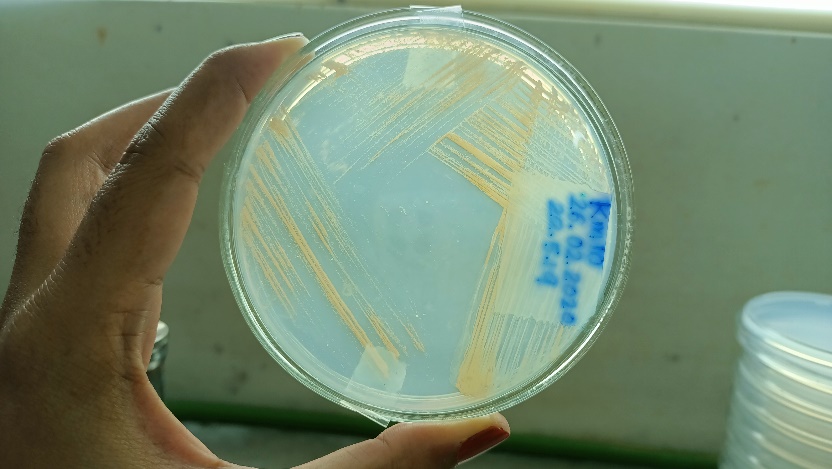 | 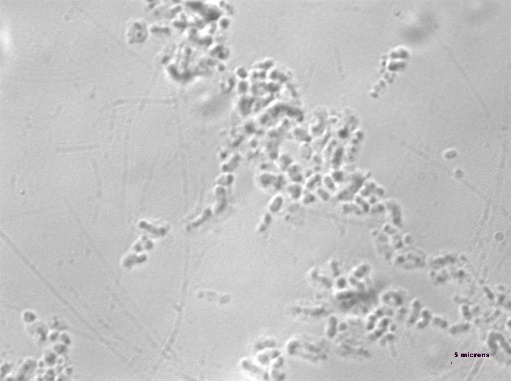 |
| **20** | Malegaon, Indrayani | IR 1 | 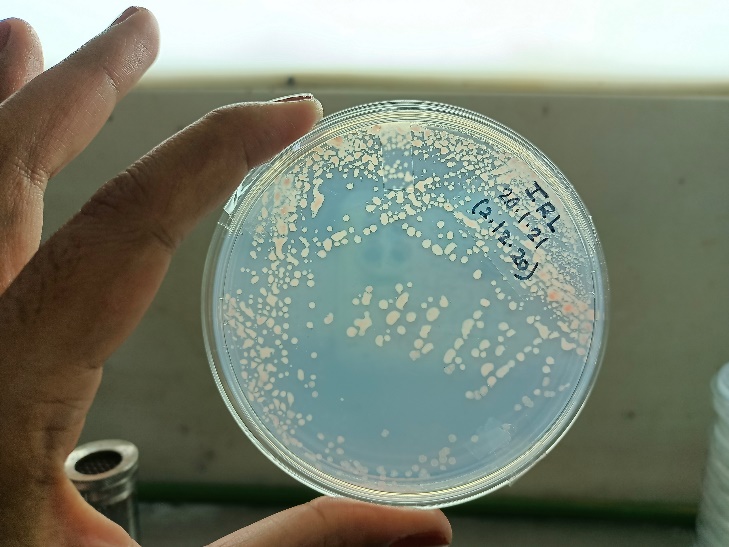 | 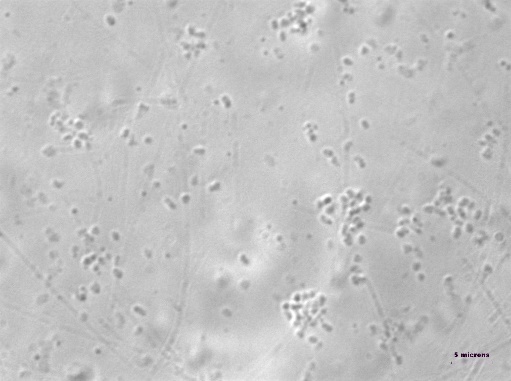 |
| **21** |  | IR 2 | *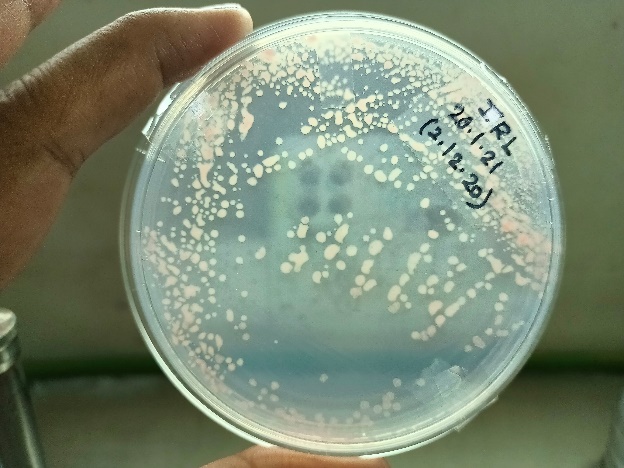* | 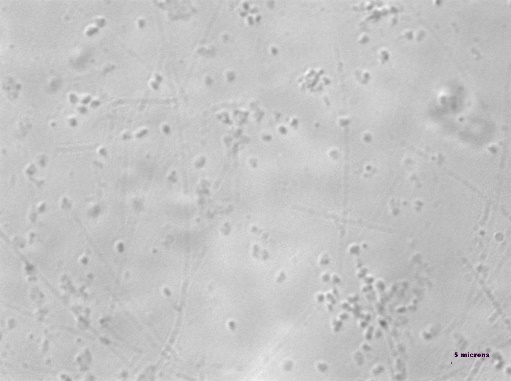 |
| **22** | Malegaon, Ratna stem | RS2 | 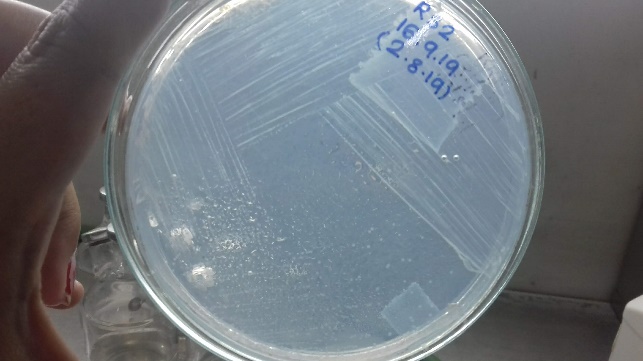 | 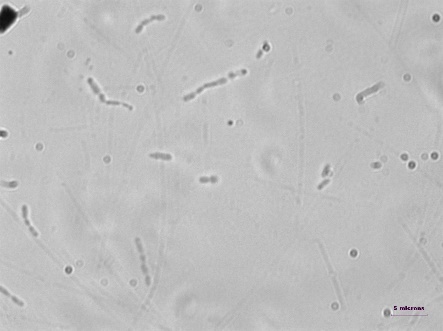 |
| **23** | Kasar Amboli, Ratna | KAR5Ro7 | 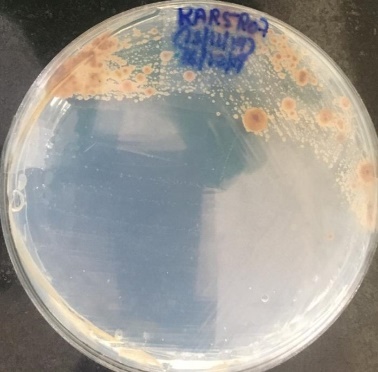 | 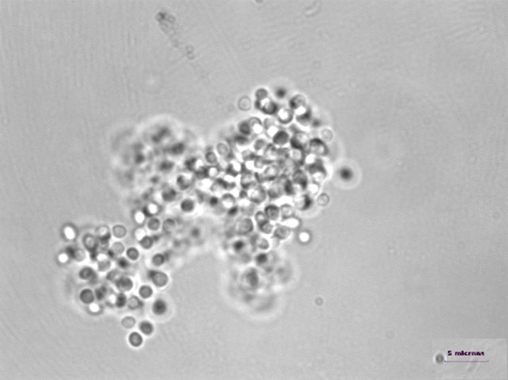 |
| **24** | Malegaon, Basmati | BM10 | 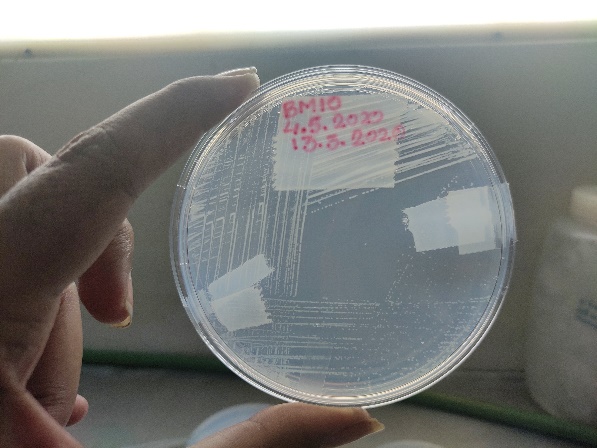 | 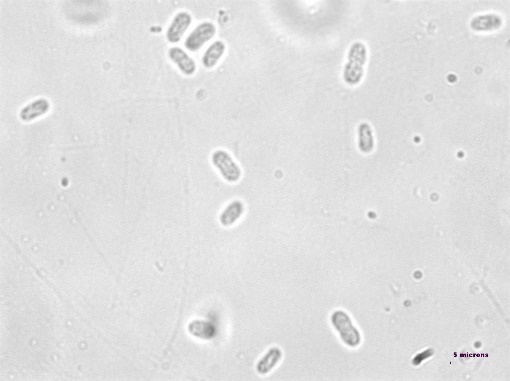 |
| **25** | Kalbhorwadi, Indrayani | Kb7 | ** | 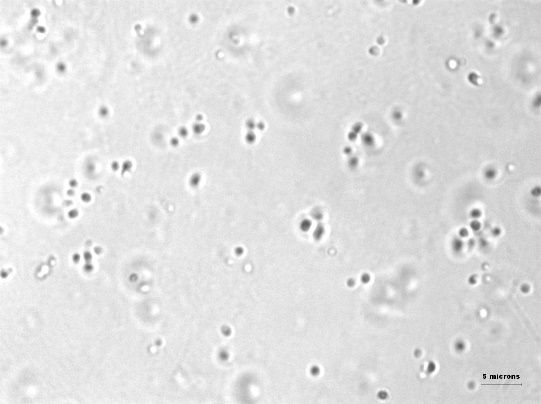 |
| **26** | Kasar Amboli | URRH | 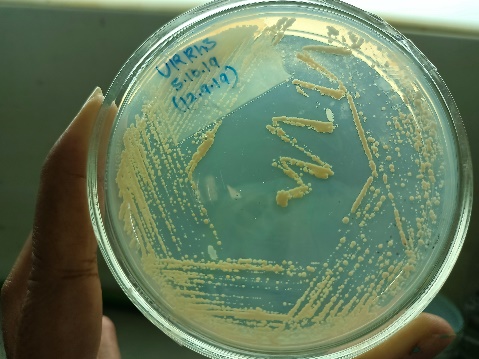 | 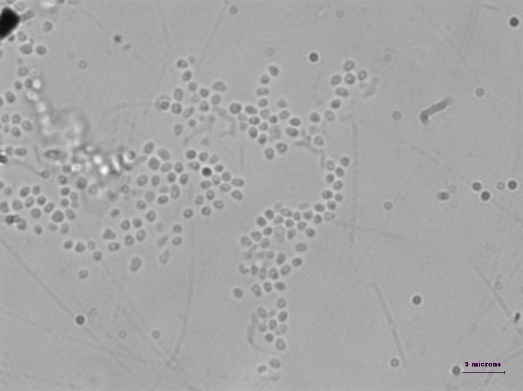 |

**Supplementary Figure 3:** Growth of isolates on solid media plates and their respective phase-contrast images with 1000X magnification. The bar represents 5 µm.


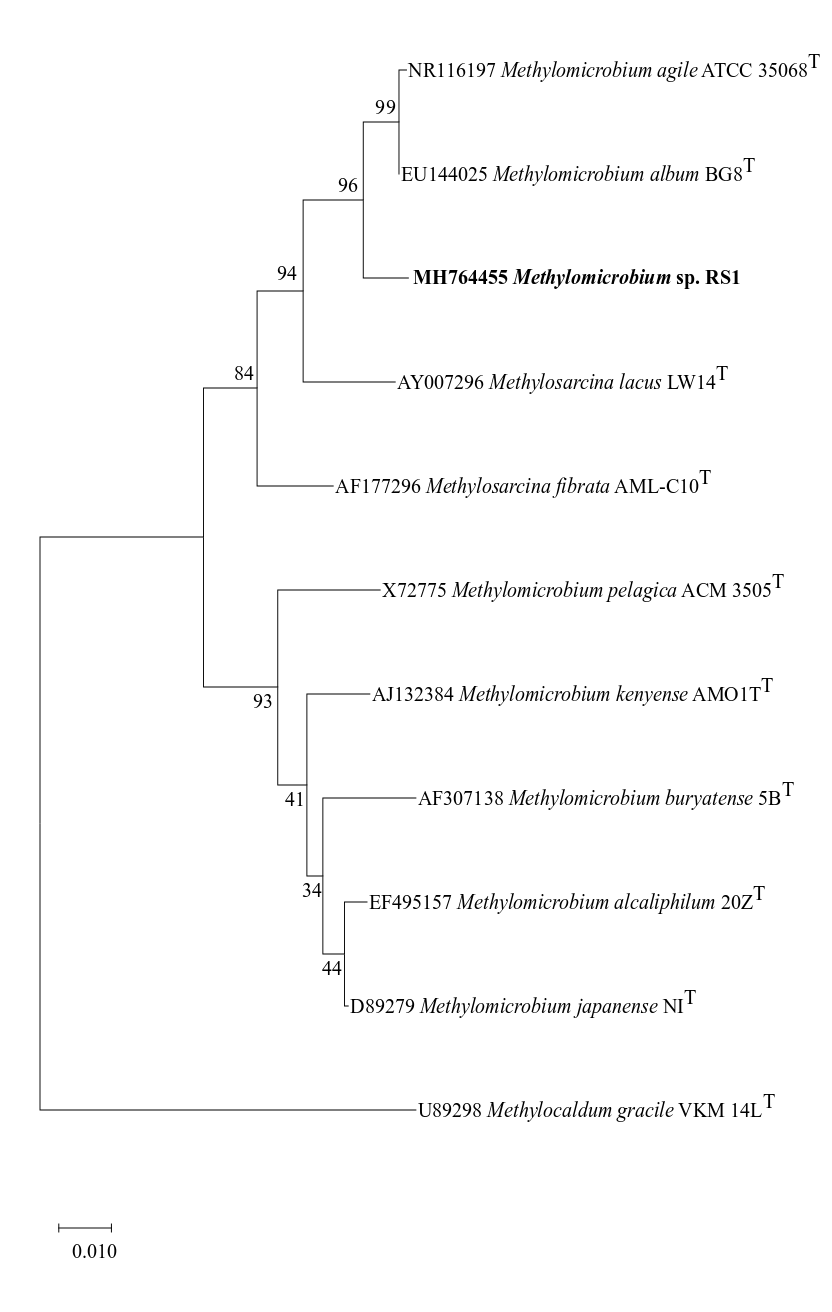
 **Supplementary Figure 4a:** Maximum-likelihood 1000 bootstrap tree of the 16S rRNA gene sequence of strain RS1 in comparison with the closely associated *Methylococcaceae* bacteria. The phylogenetic tree was constructed using MEGA X and analysed based on the Tamura-Nei model. Bar represented 1% divergence


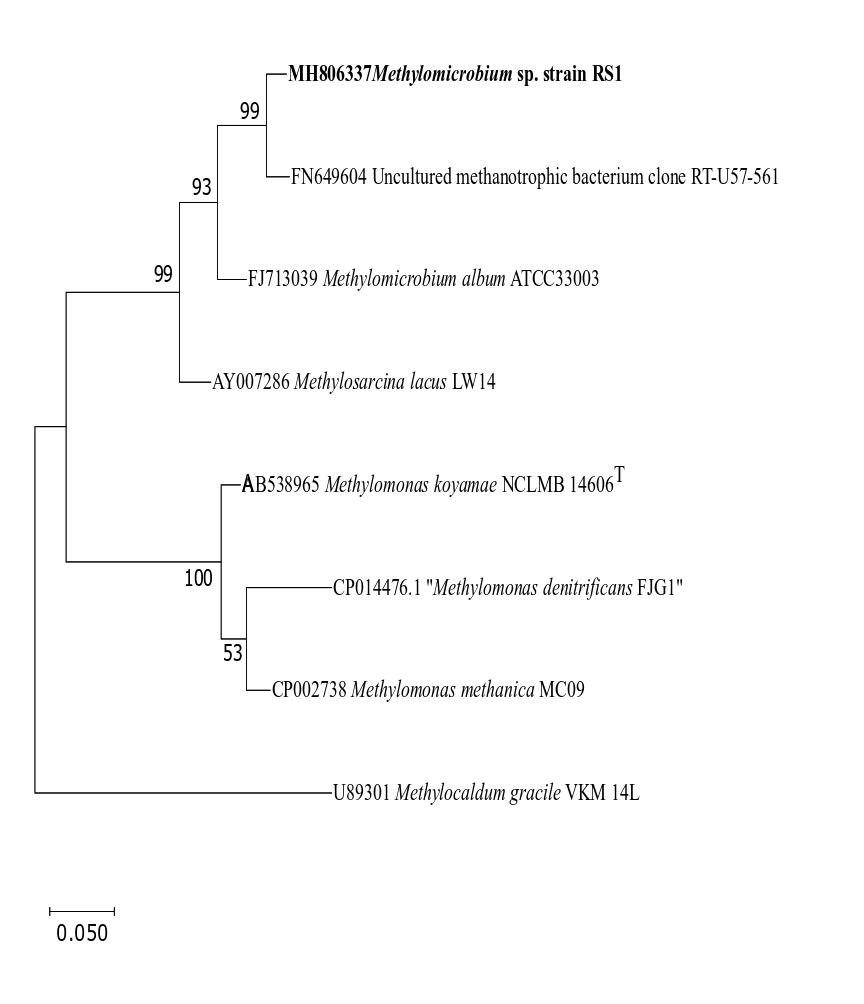


**Supplementary Figure 4b:** Maximum-likelihood 1000 bootstrap tree of the partial *pmo*A nucleotide sequence of strain RS1 in comparison with the *pmo*A gene sequences of closely related Type I methanotrophs. The evolutionary history was inferred by using the maximum-likelihood method based on the Tamura-Nei model with MEGAX. Bar representing 5% divergence.
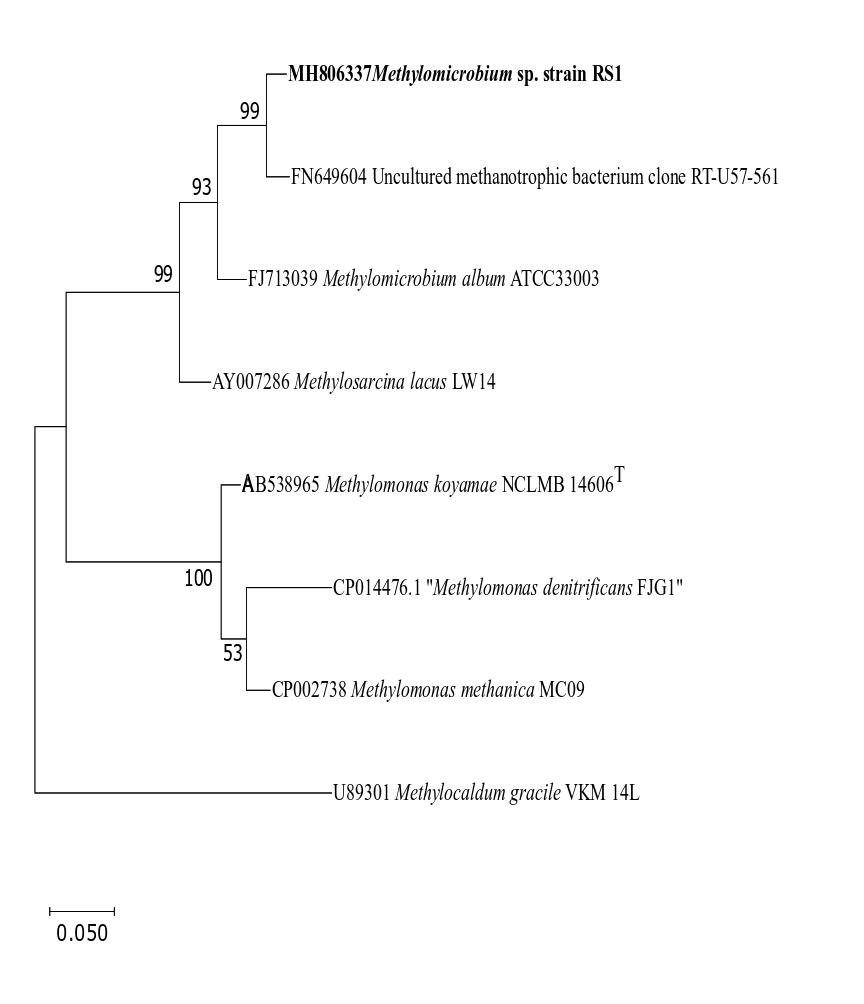
 **Supplementary Figure 4b**
