# Supplementary material for: Cultivation of Important Methanotrophs From Indian Rice Fields
Source: Front Microbiol. 2021 Sep 3;12:669244. doi: 10.3389/fmicb.2021.669244 (PMC8447245; doi:10.3389/fmicb.2021.669244)
Supplement: Supplementary Material — Growth curves and methane oxidation of the cultures. [file Data_Sheet_1.PDF]

Supplementary material: Growth curves and methane oxidation of the cultures

Analysis of methane oxidation potential of representative strains of each genus was carried, and the corresponding growth in terms of OD was measured. Growth curves with simultaneous methane oxidation curves were plotted. To determine the methane oxidation potential of isolates, dilute Nitrate Mineral Salts Medium (NMS) was used. One ml of each culture of ~0.1 OD_{600} (Optical density at 600 wavelengths) was inoculated in a sterilized 9 ml media containing serum bottles (Capacity: 35ml) with rubber butyl stoppers and sealed with aluminum seals. Five ml methane was added in 25 ml headspace, resulting in 20% methane in headspace, corresponding to 200 micromoles of methane. The experiment was set in biological replicate (n=3) along with controls. The headspace of inoculated serum bottles contains methane and air (20:80) environmental conditions. Determination of OD_{600} and methane oxidation rate was monitored throughout the experiment. Fresh air (was added to the culture after 7 days of incubation to avoid oxygen limitation.

The methane concentration was measured using Chemito 8510 Gas Chromatography, India, equipped with a flame ionization detector (FID) [Column: Porapak Q (80/100mesh, 3.2mm X2); Injector temperature: 110ºC; Detector temperature: 120ºC; Oven temperature: 100ºC; Carrier gas N_2 (15-18 ml/min), H_2 (50 ml/min), and air (250 ml/min)]. The GC peak area of the control bottles and the test bottles were compared and converted to µmoles of CH_4.

A line graph was plotted by incubation period (hours) on the x-axis and OD_{600} on the y-axis against methane concentration on the z-axis. Growth curves versus methane oxidation seen in the representative strains are shown in the figure below.

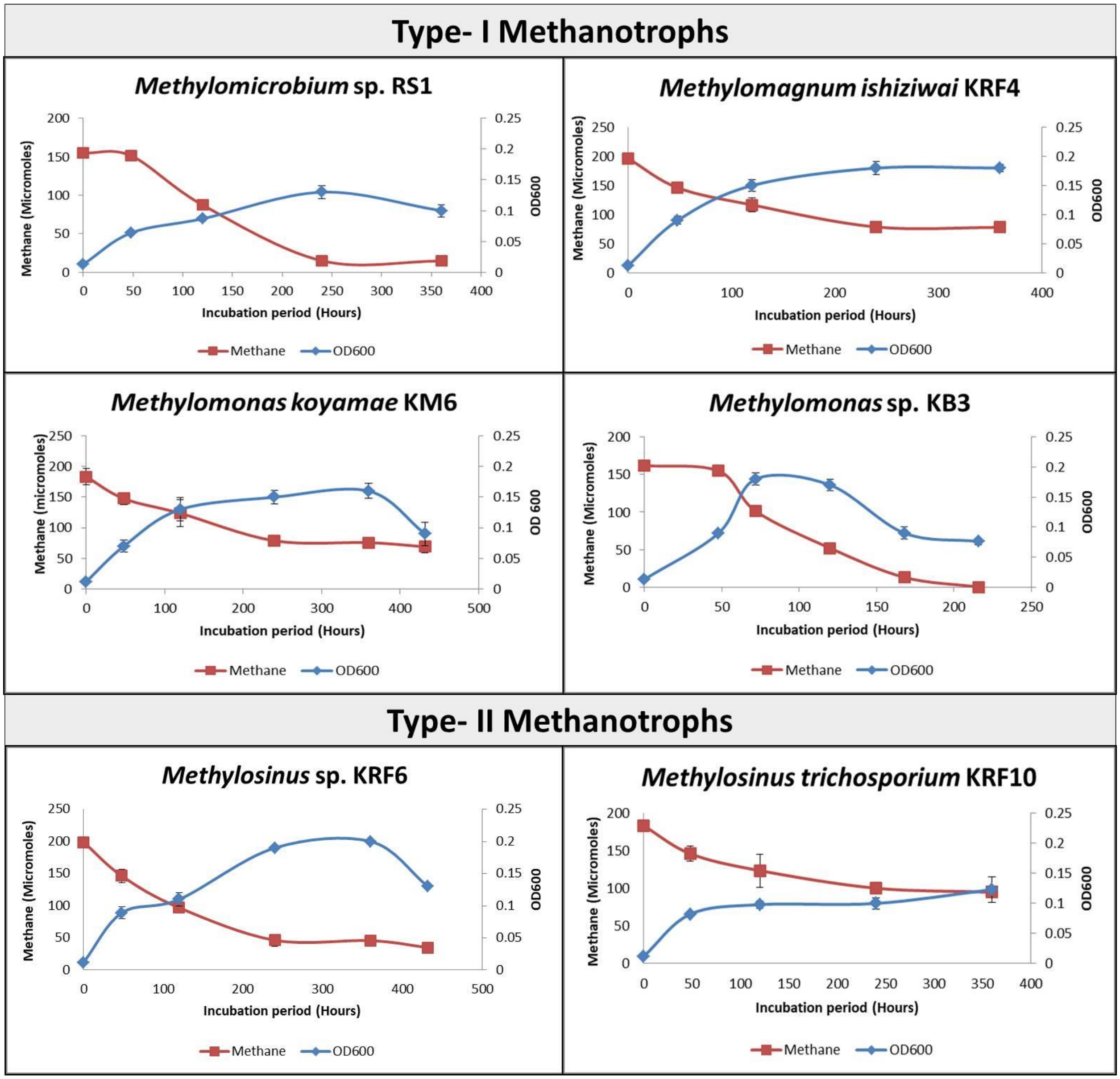

Supplementary Figure: Methane oxidation rate graphs of methanotrophs isolates: Cultures are: *Methylomicrobium* sp. RS1, *Methylomagnum ishiziwai* KRF4 and *Methylomonas koyamae* KM6, *Methylosinus* sp. KRF6, *Methylosinus trichosporium* KRF10. *Methylomonas* Kb3 (from our previous study) is shown for comparison purposes.
