# Supplementary material for: Cultivation of Important Methanotrophs From Indian Rice Fields
Source: Front Microbiol. 2021 Sep 3;12:669244. doi: 10.3389/fmicb.2021.669244 (PMC8447245; doi:10.3389/fmicb.2021.669244)
Supplement: Supplementary file 3 [file Data_Sheet_3.docx]

| **Supplementary Table 1: Details of sample location and sampling time** | | | |
| --- | --- | --- | --- |
| **S. No** | **Habitats** | **Geographical location, climate** | **Collection date, season, and waterlogging** |
|  | Rice fields, Kb | Kalbhorwadi, Near Pune, Maharashtra (18.37° N, 73.45° E) | October 2013, monsoon |
|  | Rice fields, KM (Konkan Marshy) | Chiveli, Konkan, Maharashtra (17.54° N, 73.38° E) | October 2014, monsoon |
|  | Rice field, KRF and Kerala mud sample | Alleppey, Kerala (9.49° N,76.33° E) | December 2017, winter |
|  | Rice fields | Malegaon and Kasar Amboli, Mulshi, near Pune, Maharashtra (18.50° N,73.51° E) | September 2017, monsoon |

| **Supplementary Table 2: Identification of isolates** | | | | |
| --- | --- | --- | --- | --- |
| **S. No.** | **Sample** | **Isolate name, MCMB number** | **Identification based on pmoA functional gene (blastn)** | **Percent similarity based on 16S rRNA** |
| 1 |  | KRF 2 | *Methylomonas koyamae* 97% | *Methylomonas koyamae* 99% |
| 2 |  | KRF 3 | *Methylomonas koyamae* 90% | *Methylomonas koyamae* 99% |
| 3 |  | KRF 4, MCMB- 1475 | *Methylomagnum ishizawai* 99% | *Methylomagnum ishizawai* 100% |
| 4 |  | KRF 5 | *Methylomonas koyamae* 90% | *Methylomonas koyamae* 99% |
| 5 |  | KRF 6 | *Methylosinus sporium* 91% | *Methylosinus sporium* 98% |
| 6 |  | KRF 7 | *Methylosinus sporium* 98% | *Methylosinus sporium* 98% |
| 7 |  | KRF 8 | *Methylosinus sporium* 98% | *Methylosinus sporium* 98% |
| 8 |  | KRF 9 | *Methylosinus sporium* 98% | *Methylosinus sporium* 98% |
| 9 |  | KRF 10 | *Methylosinus trichosporium* 96% | *Methylosinus trichosporium* 99% |
| 10 | Kerala rice field, mud sample | KM 1, MCMB- 1476 | *Methylomonas koyamae* 92% | *Methylomonas methanica* MC09 97% |
| 11 |  | KM 2 | *Methylomonas koyamae* 97% | *Methylomonas koyamae* 99% |
| 12 |  | KM 3 | *Methylomonas koyamae* 97% | *Methylomonas koyamae* 99% |
| 13 |  | KM 4 | *Methylomonas koyamae* 97% | *Methylomonas koyamae* 99% |
| 14 |  | KM 5 | *Methylomonas koyamae* 97% | *Methylomonas* sp. Kb3 99% [40] |
| 15 |  | KM 6 | *Methylomonas koyamae* 90% | *Methylomonas koyamae* 99% |
| 16 |  | KM 7 | *Methylosinus sporium* 98% | *Methylosinus sporium* 100% |
| 17 |  | KM 8 | *Methylosinus sporium* 98% | *Methylosinus sporium* 100% |
| 18 |  | KM 9B | *Methylocystis hirsuta* 94% | *Methylocystis hirsuta* 99% |
| 19 |  | KM 9W | *Methylocystis echinoides* 97% | *Methylocystis echinoides* 99% |
| 20 |  | KM 10 | *Methylocystis rosea* 95% | *Methylocystis rosea* 99% |
| 21 | Malegaon, Indrayani | IR 1 | *Methylomonas koyamae* 97% | *Methylomonas methanica* 97% |
| 22 |  | IR 2 | *Methylomonas koyamae* 97% | *Methylomonas methanica* 97% |
| 23 |  | IS 1 | *Methylomonas koyamae* 94% | *Methylomonas koyamae* 100% |
| 24 | Malegaon, Ratna stem | RS1, MCMB- 1473 | *Methylomicrobium agile* ATCC 35068 93.90% | *Methylomicrobium agile* ATCC 35068 97.73% |
| 25 |  | RS2 | *Methylomicrobium agile* ATCC 35068 93.82% | *Methylomicrobium agil*e ATCC 35068 98.47% |
| 26 | Kasar Amboli, Ratna | KAR5Ro7 | *Methylocaldum gracile* 99% | *Methylocaldum gracile* 99% |
| 27 | Malegaon, Basmati | BM10, MCMB- 1477 | *Methylocucumis oryzae* 96% | *Methylocucumis oryzae* 99% |
| 28 | Kalbhorwadi, Indrayani | Kb7 | *Methylocystis echinoides* 96% | *Methylocystis echinoides* 99% |
| 19 | Kasar Amboli | URRH | *Methylosinus sporium* 98% | *Methylosinus sporium* 98% |

| **Supplementary Table 3: ANIb, AAI, & DDH calculation of strain RS1 with its closely related members** | | | |
| --- | --- | --- | --- |
| **Closely related members** | **ANIb value** | **AAI value** | **DDH value** |
| *Methylomicrobium* sp. RS1 | - | - | - |
| *Methylomicrobium album* BG8 | 90.65 | 90.11 | 44.30% [41.8 - 46.9%] |
| *Methylomicrobium agile* ATCC 35068 | 90.69 | 89.82 | 44.60% [42.1 - 47.2%] |
| *Methylomicrobium lacus* LW14 | 80.80 | 79.55 | 25.30% [23 - 27.8%] |
| *Methylomicrobium kenyense* AMO | 70.03 | 62.81 | 19.20% [17 - 21.6%] |
| *Methylomicrobium buryatense* 5G | 69.46 | 62.20 | 18.00% [15.8 - 20.3%] |
| *Methylomicrobium buryatense* 5GB1C | 69.45 | 62.33 | 18.00% [15.8 - 20.3%] |
| *Methylomicrobium alcaliphilum* 20Z | 69.27 | 61.93 | 18.10% [15.9 - 20.4%] |
